# Global signalling network analysis of luminal T47D breast cancer cells in response to progesterone

**DOI:** 10.3389/fendo.2022.888802

**Published:** 2022-08-11

**Authors:** Roni H. G. Wright, Viviana Vastolo, Javier Quilez Oliete, José Carbonell-Caballero, Miguel Beato

**Affiliations:** ^1^ Center for Genomic Regulation (CRG), Barcelona Institute of Science and Technology (BIST), Barcelona, Spain; ^2^ Basic Sciences Department, Faculty of Medicine and Health Sciences, Universitat Internacional de Catalunya, Barcelona, Spain; ^3^ Universitat Pompeu Fabra (UPF), Barcelona, Spain

**Keywords:** progesterone, breast cancer, phosphoproteome, signalling, MAPK/ERK signalling, chromatin, PARylation, cell proliferation

## Abstract

**Background:**

Breast cancer cells enter into the cell cycle following progestin exposure by the activation of signalling cascades involving a plethora of enzymes, transcription factors and co-factors that transmit the external signal from the cell membrane to chromatin, ultimately leading to a change of the gene expression program. Although many of the events within the signalling network have been described in isolation, how they globally team up to generate the final cell response is unclear.

**Methods:**

In this study we used antibody microarrays and phosphoproteomics to reveal a dynamic global signalling map that reveals new key regulated proteins and phosphor-sites and links between previously known and novel pathways. T47D breast cancer cells were used, and phospho-sites and pathways highlighted were validated using specific antibodies and phenotypic assays. Bioinformatic analysis revealed an enrichment in novel signalling pathways, a coordinated response between cellular compartments and protein complexes.

**Results:**

Detailed analysis of the data revealed intriguing changes in protein complexes involved in nuclear structure, epithelial to mesenchyme transition (EMT), cell adhesion, as well as transcription factors previously not associated with breast cancer cell proliferation. Pathway analysis confirmed the key role of the MAPK signalling cascade following progesterone and additional hormone regulated phospho-sites were identified. Full network analysis shows the activation of new signalling pathways previously not associated with progesterone signalling in T47D breast cancer cells such as ERBB and TRK. As different post-translational modifications can mediate complex crosstalk mechanisms and massive PARylation is also rapidly induced by progestins, we provide details of important chromatin regulatory complexes containing both phosphorylated and PARylated proteins.

**Conclusions:**

This study contributes an important resource for the scientific community, as it identifies novel players and connections meaningful for breast cancer cell biology and potentially relevant for cancer management.

## Background

Female steroid hormones, oestrogen and progesterone play a key role not only in the normal development of target tissues during puberty, pregnancy and menopause but also in breast and endometrium cancer cell proliferation. Breast cancer cells respond to progestin exposure with two intermingled pathways that culminate in extensive gene expression changes and entry in the cell cycle. The classical view is that the hormone diffuses through the cell membrane and binds to intracellular progesterone receptors (PR), which are maintained in an inactive state by a chaperone complex, including Heat Shock Proteins 70 and 90 (HSP70/90). Upon hormone binding, PR weakens its interaction with the chaperones, dimerizes and moves to chromatin where it eventually binds to palindromic DNA sequences called progesterone responsive elements (PREs) (1). Once bound to chromatin, PR recruits various co-regulators and chromatin remodellers that modulate access for the transcription machinery including RNA polymerase II (2).

This simplified model was further complicated later by the finding that a tiny fraction of PR (3-5%) is attached to the cell membrane *via* palmitoylation at C820 (3, 4), forming a complex with estrogen receptor alpha (ERa) (5). Upon binding progestins, the membrane anchored PR activates SRC, either directly (6) or *via* ERa, initiating a kinase signalling pathway that ends in activation of the extracellular signal-regulated kinase (ERK) (5). ERK1 phosphorylates intracellular PR at S294 favoring its dissociation from the chaperone complex. In the cell nucleus ERK1 activates MSK1 (Mitogen-and stress activated protein kinase 1), resulting in the formation of a ternary complex of PR-ERK1-MSK1, which is the active form of PR able to regulate chromatin structure and gene expression. ERK also activates cyclin dependent kinase 2 (CDK2) that in turns activates ARTD1 (ADP-ribose transferase 1) by phosphorylating two serines in the NAD+ binding pocket (7). Phosphorylation contributes to dissociation of histone H1 and H2A/H2B dimers (7, 8) and to local chromatin opening by further recruitment of transcription factors, co-regulators, histone modifiers (PCA, P3000) and ATP-dependent chromatin remodellers (NURF and BAF), ultimately leading to the activation of gene expression changes (9–11). Moreover, there is also evidence for the activation by progesterone of other signaling pathways induced by progesterone, such as AKT (12), cAMP (13, 14), GSK3 (15) and STAT (16). However, many of these studies used different types of cells derived from endometrial or ovarian tissues (17, 18).

In addition to the key role of the kinase cascades, we have identified a pivotal role for another post-translational modification in progestin induced gene regulation; namely Poly-ADP-ribosylation (PARylation). As discussed above the PAR polymerase PARP1, also known as ADP-ribosyltransferase 1 (ARTD1), is activated within the initial minutes following hormone exposure *via* phosphorylation by CDK2 (7), giving rise to a large increase in PARylation within the cell nucleus (7). PARylation of ARTD1 itself and of chromatin proteins is essential for the initial dissociation of histone H1 (19, 20). We also found that degradation of PAR to ADP-Ribose by PAR glycohydrolase (PARG) is required for complete chromatin remodelling and activation of the gene expression network (21). Mass spec analysis of the proteins interacting with PAR in T47D cells exposed to progestins revealed structural proteins, DNA damage response proteins and chromatin modifying enzymes (21). One key enzyme identified in this study was NUDT5 or NUDIX5 (Nudix hydrolase 5), which hydrolyses ADPR to AMP and ribose-5-phosphate. Subsequently, we found that upon dephosphorylation at T45, NUDT5 can use ADPR and diphosphate for the synthesis of ATP (21). In this way, part of the ATP consumed during the synthesis of NAD^+^ and stored in PAR is recovered and used for chromatin remodeling and changes in gene expression. The synthesis of nuclear ATP is transient, peaking at 40 minutes after hormone exposure and returning to basal levels after ~60 minutes. However, although we know that nuclear ATP synthesis is essential for the initial chromatin remodelling, the role of the nuclear ATP at later time points is unclear. We can envision several hypotheses; as a direct, local source of ATP for the massive amount of ATP-dependent chromatin remodelling and 3D conformational changes induced by hormone (9, 22) or to facilitate phase separation of chromatin fiber (23). In any case, we know that nuclear ATP synthesis by NUDT5 is essential for the generation and maintenance of the cancer stem cell population (24).

Over the past years, there has been a large number of studies investigating the role and mechanism of action of one or more of the pathway components in response to progesterone exposure, revealing a dynamic crosstalk between canonical pathways (25–28). However, these studies focus on one or very few components at a time, and do not explain how these various pathways interact and coordinate the cell response to hormones. The work described here aims to provide a more comprehensive map of progesterone signalling in breast cancer using luminal A T47D breast cancer cells as a model, combining antibody arrays technology, shotgun proteomics, and previously published PARylation datasets to develop for the first-time a global map of the dynamic signalling events induced by progestins in T47D breast cancer cells.

## Methods

### Cell culture

The hormone receptor positive breast cancer cell line T47D^M^ (CLS Cat# 300353/p525_T-47D, RRID : CVCL_0553) was used in all experiments unless otherwise stated. T47D^M^ cells were routinely grown in RPMI (Supplemented with 10% foetal bovine serum (FBS), penicillin/streptomycin (pen/strep), L-glutamine (L-glut) as previously described (21). For hormone induction experiments, cells were seeded at a concentration of 5 x 10^6^ per 150mm cell culture dish in RPM1 white (15% charcoal stripped FBS, Pen/strep, L-glut) for 48 hours. 16 hours prior to hormone induction for time indicated (10nM R5020), the medium was replaced with RPM1 white (0% FBS, Pen/strep, L-glut). Samples were harvested at the time points indicated.

### BCA assay

The total protein content of the samples was calculated prior to antibody array, mass spec or western blotting analysis using BCA assay (Thermo Fisher, catalogue number 23227) according to manufacturer’s instructions.

### Protein visualization

Changes in phosphorylation sites within individual proteins identified was confirmed by western blotting as previously described (19) using specific antibodies; Progesterone receptor (PGR) phospho-S162 (Abcam Cat# ab58564, RRID : AB_883089), and as a loading control, total PGR (Santa Cruz Biotechnology Cat# sc-7208, RRID : AB_2164331), total CDK2 (Santa Cruz Biotechnology Cat# sc-6248, RRID : AB_627238) or CDK2 phospho-T160 (Abcam Cat# ab47330, RRID : AB_869087).

### Antibody microarray

Phosphorylation antibody array analysis was carried out by Kinexus™ using Kinexus ™ Antibody Microarray (KAM) technology. For each time point 3 biological replicates were prepared independently. For each replicate, 50ug of protein lysate was prepared and samples prepared by Kinexus™ in house (Kinexus Bioinformatics Corporation, RRID : SCR_012553). Signal quantification was performed using ImaGene 8.0 (ImaGene, RRID : SCR_002178) from BioDiscovery (BioDiscovery, RRID : SCR_004557). Background corrected raw intensity data was logarithmically transformed with base 2 and Z scores calculated (29). Any poor-quality spots based on morphology and/or background, were flagged as unreliable and removed from any subsequent analysis.

### Mass spec sample preparation

Samples (triplicates from independent experiments, per time point) were precipitated with 6 volumes of cold acetone and the pellet was dissolved in 6M Urea/200mM ammonium bicarbonate at a concentration of 1 µg/µL. 260 µg of each sample was reduced with dithiothreitol (DTT, 10mM, 37°C, 60 min) and alkylated with iodoacetamide (IAM, 20mM, 25°, 30 min). The resulting protein extract was first diluted to 2M urea with 200 mM ammonium bicarbonate for digestion with endoproteinase LysC (1:10 w:w, 37°C, o/n, Wako, cat # 129-02541), and then diluted 2-fold with 200 mM ammonium bicarbonate for trypsin digestion (1:10 w:w, 37°C, 8h, Promega cat # V5113). Digested peptides were subjected to phosphopeptide enrichment using the “TiO2 Phosphopeptide Enrichment and Clean-up Kit (Pierce)”

45% of each enriched sample was analyzed using an Orbitrap Fusion Lumos with an EASY-Spray nanosource coupled to a nano-UPLC system (EASY-nanoLC 1000 liquid chromatograph) equipped with a 50-cm C18 column (EASY-Spray; 75 µm id, PepMap RSLC C18, 2 µm particles, 45 °C). Chromatographic gradients started at 5% buffer B with a flow rate of 300 nL/min and gradually increased to 22% buffer B in 105 min and to 32% in 10 minutes. After each analysis, the column was washed for 10 min with 95% buffer B (Buffer A: 0.1% formic acid in water. Buffer B: 0.1% formic acid in acetonitrile).

The mass spectrometer was operated in data-dependent acquisition mode, with full MS scans over a mass range of m/z 350–1500 with detection in the Orbitrap (120K resolution) and with auto gain control (AGC) set to 100,000. In each cycle of data-dependent acquisition analysis, following each survey scan, the most intense ions above a threshold ion count of 10,000 were selected for fragmentation with HCD at normalized collision energy of 28%. The number of selected precursor ions for fragmentation was determined by the “Top Speed” acquisition algorithm (maximum cycle time of 3 seconds), and a dynamic exclusion of 60 s was set. Fragment ion spectra were acquired in the ion trap with an AGC of 4000 and a maximum injection time of 300 ms.

Raw files, quantified peptides, proteins and phosphosites (5% FDR) are deposited and publicly available in the UCSD Data depository, MassIVE (Project ID: MSV000089820, ftp://MSV000089820@massive.ucsd.edu).

### Phosphopeptide analysis

Acquired data were analyzed using the Proteome Discoverer software suite (v2.0, Thermo Fisher Scientific), and the Mascot search engine (v2.5.1, Matrix Science) was used for peptide identification. Data were searched against a Human protein database derived from SwissProt plus the most common contaminants. A precursor ion mass tolerance of 7 ppm at the MS1 level was used, and up to three missed cleavages for trypsin were allowed. The fragment ion mass tolerance was set to 0.5Da. Oxidation of Methionine, N-terminal protein acetylation and phosphorylation in Serine, Threonine and Tyrosine were defined as variable modification and carbamidomethylation of Cysteines was set as fixed modification. The identified peptides were filtered by 5%FDR. Peptide areas were obtained with the area under from extracted ion chromatogram using the “Precursor Ions Area Detector” node from Proteome Discoverer.

### Bioinformatic procedures

Gene Ontology (GO) GO-Biological process (GO-BP), GO-Molecular Process (GO-MF), KEGG (KEGG, RRID : SCR_012773) and Biocarta (BioCarta Pathways, RRID : SCR_006917) pathway analysis of networks was carried out using GeneMania application (GeneMANIA, RRID : SCR_005709) (30) within Cytoscape (Cytoscape, RRID : SCR_003032). Clustering analysis, similarity analysis was carried out using GeneE. Network analysis was performed using Cytoscape v3.5 (31). The initial prior knowledge network (PKN) was generated based on known protein-protein interactions only validated experimentally. Significantly enriched pathways were analyzed within the network using CytoKEGG application within Cytoscape. The parent network and each of the individual pathway networks are available for visualisation and further analysis (cytoscape session link **Supplementary Materials**). Comprehensive resource of mammalian protein complexes (Corum analysis) was carried out using online tool http://mips.helmholtz-muenchen.de/corum/CORUM, RRID : SCR_002254 (Giurgiu et al., 2018). Functional classification GO biological process (BP), molecular function (MF) and cellular component (CC) were carried out using molecular signatures database (MSigD) within Gene Set Enrichment (GSEA) tool (Gene Set Enrichment Analysis, RRID : SCR_003199) and terms with a p value of less than 0.001 were considered significantly enriched (32, 33).

### Kaplan Meyer and protein expression in clinical samples

Analysis of the overall survival of breast cancer patients using a Kaplan-Meier plot were carried out using KMPlotter (34), https://kmplot.com/analysis/to assess the correlation between the expression of the gene (mRNA) and survival in 4929 breast cancer patient samples. Sources for the databases include GEO, EGA, and TCGA. All patients’ samples were included in the analysis shown, i.e ER, PR status, subtype, lymph node status and grade. Analysis of protein expression levels in breast tumor versus normal samples were representative of those within the Human Protein Atlas database (35) http://www.proteinatlas.org.

## Results

### Prior knowledge network

Before starting to add quantitative dynamic data to the already existing knowledge of progesterone signalling events in T47D cells, we have generated a “prior knowledge network (PKN)”, based on the published literature (**Figures S1A, B**, and **Supplementary Material; Cytoscape Session 1**). Each protein-protein interaction is characterized based on type (interaction, phosphorylation or dissociation) and is displayed as a unique edge. The corresponding literature is given in **Figure S1B** and within the Cytoscape session. The PKN already shows the key role played by kinases in response to progestins. First, progestins *via* ERa activate SRC1 that phosphorylates MAPKK1, that activates ERK1, that phosphorylates PGR, resulting in dissociation from the HSP90A and B proteins (36, 37). Activated ERK1 also phosphorylates ERa at S118 (38). ERK1 in association with hormone receptors translocates to the cell nucleus where it phosphorylates MSK1 (39), leading to the formation of an active complex PR-ERK-MSK1 that interacts with chromatin containing accessible PRE. Activated PR also interacts with PLK1 that activates MLL2 (40), with CDK2 that phosphorylates and activates ARTD1 (7), and with JAK2 that activates STAT5 (16). Simultaneously, membrane activated SRC1, also activates RAS and EGFR (41), which feeds back activating the MAPK cascade. Membrane associated ERa also activates PI3K and cAMP, which upon binding with AKT and PKA respectively lead to the activation (via interaction and direct phosphorylation) of GSK3, mTOR (42, 43) and the arginine methyltransferases CARM1 and PRMT1 within the nucleus (44–46). This brief description of the PKN shows that it already encompasses a great degree of complexity and complementary connections that need additional data to be resolved.

### Microarrays of antibodies to phosphorylated sites in proteins

**Figure 1:**
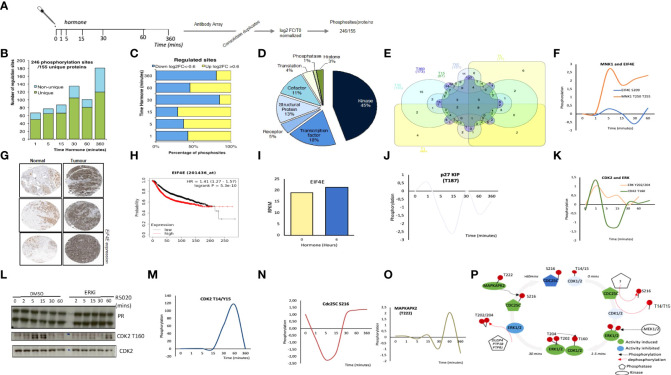
Targeted Antibody Array Phosphorylation data following hormone. **(A)** Schematic overview of experimental procedure. Synchronized T47D cells were exposed to hormone for the length of time indicated. Triplicate samples were harvested and phosphorylated proteins were identified using an antibody microarray (see materials and methods). Data was log_2_ normalized resulting in a total of 246 significant phosphosites from 155 unique proteins. **(B)** Number of regulated sites per time point, log_2_FC (Fold change) >0.6<-0.6 versus time 0. **(C)** Breakdown of up (>0.6log_2_FC) and down (<-0.6 log_2_ FC) per time point versus T0. **(D)** Functional classification of the proteins identified as significantly phosphorylated across all time points, individual time point functional analysis (per time point see **Figure S2C**). **(E)** Venn diagram showing the overlap of significantly regulated phosphorylation sites across all time points. **(F)** Log_2_ FC following hormone of phosphorylated Mnk1 (T197/202) and EIF4E S209. **(G)** Expression of EIF4E in normal versus breast tumor samples from breast cancer patients (Protein Atlas, see materials and methods). **(H)** Kaplan Meyer overall survival stratifying patients based on the expression level of EIF4E in breast cancer data set (p=5.3e-10). **(I)** mRNA expression level of EIF4E in T47D cells treated with hormone. Dynamics of **(J)** p27/KIP T187 **(K)** CDK2 T160, ERK Y202/204. **(L)** Expression level of total PR and CDK2 and phospho-CDK2 T160 in T47D breast cancer cells exposed to hormone in the presence or absence of ERK inhibitor (ERKii) as determined by western blotting using specific antibodies. **(M)** CDK2 T14/Y15 phosphorylation in response to hormone as determined by antibody array. Dynamics of **(N)** Cdc25C S216 and **(O)** MAPKAPK2 T222 phosphorylation in response to hormone as determined by antibody array. **(P)**. Model for CDK2/ERK dynamic activation and deactivation in response to hormone based on the data presented in **(J–O)**.

Our plan was to combine antibody microarray technology and shotgun phosphoproteomics in T47D^M^ breast cancer cells exposed to 10 nM R5020 for different lengths of time, as previously described (7). For each experimental approach and exposure time total protein extracts were harvested in triplicate. For the antibody arrays, data was collected, filtered for quality control and summarized as log_2_ ratio over time zero (as described in materials and methods, **Figure S2A**). This dataset provided 246 unique phosphorylation sites corresponding to 155 proteins (**Figure 1A**; **Supplementary Table S1**). The majority of proteins contain 1 phosphorylation site, although for several proteins (Tau, RB1, MAP2K1, PTK2 and the protein kinase RPS6KA1) 7 or more significantly regulated phosphorylation sites were identified (**Figure S2B**).

Analysis of the number of phosphorylation sites clearly showed a rapid activation already 1-minute following hormone (68 significant phosphorylation events **Figure 1B**). Signalling persists throughout the time course showing two peaks at 30- and 360-minutes following hormone (**Figure 1B**). Phosphorylation sites were characterized as up or down-regulated, using a threshold for the log_2_ fold change with respect to time 0 of -0.6< or >0.6 respectively (**Figure 1C**). We see a trend for early phosphorylation sites to be dynamically increased compared to time zero, in contrast to later time points where protein phosphorylation sites as a whole decrease compared to time zero (**Figure 1C**). The majority of phosphorylation sites identified belong to protein kinases (45%), co-factors (11%), transcription factors (18%) and structural proteins (13%). (**Figure 1D**). Combining the identified phosphorylation sites over the time course reveals that the majority of sites are regulated at more than one time point (**Figure 1E**), however the protein function enrichment does not alter significantly over time, with kinases and transcription factors being the main protein groups where the phosphorylation sites are observed (**Figure S2C**).

**Figure 2:**
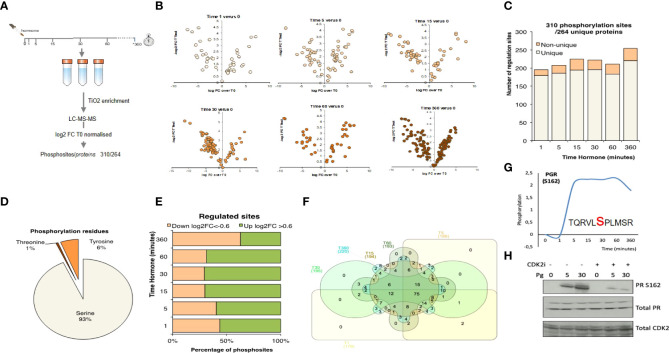
Phosphosite enriched Shotgun Proteomics following hormone. **(A)** T47D cells were treated with hormone at the times indicated. Biological triplicates were enriched for phosphopeptides using TiO2 followed by LC-MS-MS peptide identification. Data was log_2_ normalised resulting in a total of 310 phosphosites from 264 unique proteins. **(B)** Volcano plots showing phosphopeptide log_2_FC versus p-value for each of the time points following hormone. **(C)** Number of significant phosphosites identified per time point. **(D)** Analysis of the proportion of threonine, tyrosine and serine phosphorylated residues identified. **(E)** Breakdown of up (>0.6log_2_FC) and down (<-0.6 log_2_ FC) per time point versus T0. **(F)** Venn diagram showing the overlap of significantly regulated phosphorylation sites over time. **(G)** Phosphorylation of progesterone receptor PR (S162), following progesterone validated by western blotting in presence or absence of CDK2 inhibitor **(H)**. Total PR levels are shown as a loading control.

Pathway and gene ontology (GO) for biological function (BP) molecular function (MF) analysis revealed a significant increase in Cancer pathways (**Figure S2D**), signal transduction, biopolymer metabolic process and kinase activity (**Figures 2SE and F**). Within this dataset we observed a strongly upregulated phosphorylation of the MAPK Signal-Integrating Kinase 1, MNK1 at T250/T255 (**Figure 1F**) in T47D in response to progesterone stimulation. Phosphorylation of MNK1 at T250/T255 by ERK induces the activity of MNK1 (47). Once activated, MNK1 phosphorylates its targets, including the proto-oncogene Eukaryotic Translation Initiation Factor 4E (EIF4E), for which we also observed a modest phosphorylation which follows a similar pattern to MNK1 (**Figure 1F**). Activation of MNK1 has been shown to promote cell proliferation thus MNK1 inhibitors appear as an exciting opportunity for cancer therapy. MNK1 signalling play a key role in invasive breast cancer growth (48), MNK1 inhibitors have been shown to block breast cancer proliferation in multiple cell lines (49), and its downstream target EIF4E is overexpressed in tumor versus normal samples from breast cancer patients (**Figure 1G**) and associated with a poor overall survival (**Figure 1H**). Our results are the first indication that MNK1 activation may be relevant for progesterone induced breast cancer cell proliferation.

CDK2 plays an important role in progesterone signalling, activating ARTD1, and phosphorylating histone H1 (7). CDK2 activity is controlled by the formation of an active complex with the cyclin partner; either Cyclin E or A. In addition to binding the cyclin partner, CDKs are also controlled *via* interactions with Kinase Inhibitory Proteins (KIPs). p27/KIP is rapidly dephosphorylated at T187 in response to hormone, dropping sharply at 1 minute after hormone exposure (**Figure 1J**), when CDK2 is phosphorylated and activated. Phosphorylation of p27 at T187 results in its ubiquitination and degradation and inhibits the interaction with CDK2 (50), which would result in the release of CDK2 from the inhibitory protein complex resulting in the activation of ARTD1 and subsequent nuclear effects. In addition, we observe the coordinated activation of the upstream kinase of CDK2 at T160; ERK at Y202/204 (**Figure 1K**) and could validate the phosphorylation of CDK2 T160 and its dependence on ERK activity by western blotting in the presence of ERK inhibition (**Figure 1L**). CDK2 at T160 is the active phosphorylation site of CDK2 peaking at 1-minute following hormone exposure (**Figure 1K**) in contrast to the inactive phosphorylation site of CDK2 (T14/Y15) which peaks at 60 minutes following hormone exposure to silence the kinase (**Figure 1M**). The activity of the phosphatase CDC25C is key for the removal of the inhibitory T14/Y15 phosphorylation sites of CDK2. The phosphatase itself is inactivated by phosphorylation at S216. We observe a peak in CDC25C phosphorylation prior to and following 60 minutes of hormone exposure, which would permit the phosphorylation of the inhibitory phosphorylation site in CDK2 (**Figures 1M, N**). Going one step further within this pathway; MAPKAPK2, the kinase which phosphorylates CDC25C at S216 is activated following the same time dynamic as its target (**Figure 1O**). Although the importance of CDK2 in progestin induced cell proliferation has been studied (7, 51) the complex mechanism of CDK2 activation; phosphorylation of active/inactive marks, activation and regulation of upstream phosphatases and kinases was not clear until now (**Figure 1P**). These examples of the dynamic phosphorylation of MNK1 and CDK2 highlight the insight that can be gained by this type of global signalling datasets.

### Shotgun phosphoproteomics

To complement the microarray dataset, we performed shotgun phosphoproteomic analysis using mass spec. Phospho-peptides from T47D^M^ cells exposed to 10 nM R5020 for the same duration as in the array experiments, were enriched using TiO2 and phosphorylated peptides identified by LC-MS-MS (**Figure 2A**; **Figure S3A**; **Supplementary Table S1**, **S2**). We identified 7482 phosphopeptides with a FDR 5% (**Table S2**) and dynamic, significant changes in 310 unique phosphorylation sites within 264 unique proteins (**Figures 2B, C**). The majority of proteins exhibited regulation of a single phosphosite, except for the serine/arginine repetitive matrix protein, SRRM1, involved in mRNA processing and the TP53 enhancing protein TP53BP1, that exhibited 8 and 10 regulated phosphorylation sites respectively (**Figure S3B**). Most phosphorylation sites identified were phosphor-serine consistent with the biological ratio of residue specific phosphorylation (**Figure 2D**). Over the time course, changes at each time point were identified as either up (log_2_FC>0.6) or down (log_2_FC<-0.6) regulated (**Figure 2E**). Up-regulated sites prevailed at early time points and many of these phosphorylation sites were significantly regulated at more than one time point (**Figure 2F**). Pathway and GO-BP (Biological Process) and MF (Molecular Function) enrichment analysis was consistent with the antibody array enrichment and revealed an increase in pathways in cancer, biopolymer metabolic process and kinase activity (**Figures S3C–E**).

PR S294 is rapidly phosphorylated in response to hormone resulting in its activation and dissociation from chaperone complexes and increased protein turnover (52). In recent years it has been shown that clinical samples assigned as “PR low” actually have elevated levels of phosphorylated PR S294 and that this phosphorylation is associated with a genetic signature linked to cancer stem cell growth and increased recurrence which may have implications for the treatment of PR low patients with anti-progestins (53). Phosphorylation of PR S162 in the hinge region showed a strong hormone induced increase by mass spec (**Figures 2G, H**). Phosphorylation within this region of PGR has been previously reported to be mediated by CDK2 (54), which we were able to confirm as the specific phosphorylation of S162 PR in response to progesterone was strongly decreased in the presence of CDK2 inhibition (**Figure 2H**).

### Bioinformatic functional analysis

**Figure 3:**
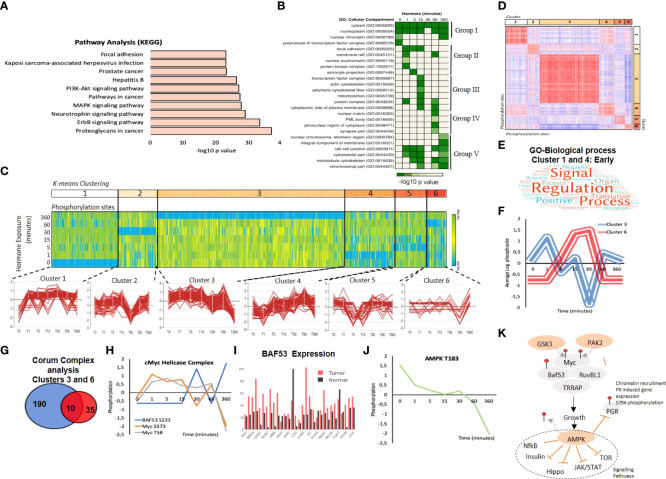
Combining Target Antibody Arrays and Shotgun Phosphoproteomic datasets following hormone. **(A)** KEGG pathway enrichment analysis of proteins identified as regulated by phosphorylation in response to hormone. **(B)** Cellular component analysis of phosphosites enriched per time point. Showing the hormone induced phosphorylation of the nucleoplasm and cytosol across all time points (group I) the activation of membrane raft proteins enriched at 1 minute (Group II) and phosphorylation of mitochondrial proteins enriched at 15 minutes (Group III), activation of nuclear structures; PML bodies and the nuclear matrix at 60 minutes (Group IV) and the activation of the cell-cell junctions and microtubules at 360 minutes (Group V). **(C)** K mean clustering of all significantly regulated phosphorylation sites over time reveals 6 distinct clusters. **(D)** Similarity matrix of clusters 1-6 reveals similar dynamics for clusters 1 and 4 and an opposing similarity in phosphorylation dynamics for clusters 3 and 6. Red indicates highly similar, well correlated, blue inversely correlated patterns of regulation. **(E)** Word cloud showing the enrichment of GO-biological processes associated with proteins identified in similar clusters 1 and 4 “Early risers” which are regulated rapidly after hormone. **(F)** Graph showing the opposing phosphorylation dynamic of proteins within clusters 3 and 6. **(G)** Venn diagram showing the overlap of significantly identified Corum protein complexes identified in clusters 3 and 6. **(H)** Phosphorylation dynamic in response to hormone of Myc S373, and T58 and BAF53 S233. **(I)** Expression level of BAF53 in tumor versus normal tissue within the TGCA dataset. **(J)** Phosphorylation of AMPL T183 decreases rapidly in response to hormone. **(K)** Model showing the key role of AMPK dephosphorylation in response to hormone in breast cancer cells, AMPK dephosphorylation is required in order for subsequent signalling cascades including NFkB, insulin, Hippo, JAK/STAT and mTOR to continue and the phosphorylation of PR S294 to take place.

In order to investigate the dynamics of progestin signalling over time and with the aim of avoiding inherent biases generated from either technical approach, we combined the significantly regulated phosphorylation sites from both datasets (**Figures 1**, **2**) resulting in a list of 420 unique phosphorylation sites within 390 proteins (**Figure S4A**). PCA analysis of the samples reveals a clear separation of the phosphorylation data at 6 hours following the hormone, given the majority of phosphorylation sites are rapid, this separation of the latest time point may reveal changes in protein abundance at this time point. The majority of these proteins showed the regulation of a single phosphorylation event with the exception of several highlighted proteins, including FAK, MAPT and EGFR (**Figure S4C**). As in the individual analysis, phosphorylation sites were significantly regulated over several time points (**Figure S4D**) and showed a switch from up-regulated sites early after hormone exposure to down-regulated sites at later time points (**Figure S4D**). KEGG pathway analysis shows a significant enrichment in MAPK, PI3K-AKT, neurotrophin (TRK) and ERBB signalling pathways (**Figure 3A**; **Supplementary Table S2**), in addition to pathways key in the progression of cancer, specifically cancer stem cells, such as focal adhesion (**Figure 3A**).

GO cellular component analysis reveals a dynamic pattern of specific cellular compartments over time (**Figure 3B**; **Supplementary Table S3**). As expected, over the whole-time course, proteins are mainly found within the cytosol and nucleoplasm. However, prior to hormone exposure, phosphorylated proteins are enriched in RNA transcription repression complex and nuclear chromatin. The addition of hormone rapidly induces the phosphorylation of the membrane rafts, components of focal adhesion and protein kinases consistent with published works whereby signalling initiates from the plasma membrane. This transient phosphorylation of the membrane rafts diminish after 1 minute and is followed by the phosphorylation of transcription factors and proteins within the cytoskeleton (**Figure 3B**). Interestingly, in line with our findings showing the generation of nuclear ATP synthesis independent of mitochondrial supplementation at 30 minutes after hormone we observe an enrichment in phosphorylated proteins located within the mitochondrial membrane at 15 minutes (**Figure 3B**). The dynamic regulation of these proteins; CYB5B (cytochrome b5), the transcriptional activator ATF2 (Cyclic AMP-dependent transcription factor ATF-2), RPS6KB1 (Ribosomal protein S6 kinase beta-1) and PI4KB (Phosphatidylinositol 4-kinase beta) (**Figure S4F**) may suggest an as yet undiscovered crosstalk between the nuclear and mitochondrial ATP synthesis pathways. Mitochondrial PR (PR-M) is a truncated isoform of the nuclear progesterone receptors PRB and PRA, which lacks the N-terminal DNA binding domain present in PRA and PRB but does contain the hinge region responsible for dimerization and the ligand binding domain (55). PR-M has been shown to increase cellular respiration hence cell energy levels in response to ligand in various physiological situations and animal models (56). Therefore, the coordinated phosphorylation of proteins within the mitochondria in response to ligand (**Figure S4F**) may provide an interesting insight into a possible crosstalk between mitochondrial PR-M and the nuclear receptors PRA and PRB.

At 60 minutes following hormone exposure the main localization of phosphorylation changes and shifts again to nuclear matrix proteins and proteins found within distinct regions of the nucleus, such as PML bodies (**Figure 3B** group IV), which may be involved in the reorganization of chromatin in response to progestins (22). At 6 hours following hormone exposure cells enter the early stages of the cell cycle and cellular movement is increased. This is also evident by the enrichment of phosphorylation sites in proteins within cell-cell junctions, the cytoskeleton and microtubules (**Figure 3B** group V).

### Protein class analysis

The majority of identified phosphorylated proteins (60%) were assigned to one class, however due to the promiscuous nature of enzymes nearly 40% were assigned to more than one class (**Figure S4G**). Taking only the parent class into account, we observed 5 distinct functions; 1) nucleic acid binding, 2) enzymes, 3) structural proteins, 4) protein modulators, and 5) proteins involved in signalling, membrane and cell-cell contacts (**Figure S4H**). Each function class consists of sub-groups (**Figures S5A–F**). The Nucleic Acid binding class includes DNA binding proteins, helicases, nucleases and RNA binding protein subgroups (**Figure S5A**). The enzyme class is dominated by kinases but also includes histone modifying enzymes, hydrolases, ligases and oxidoreductases (**Figure S5D**). The structural class is dominated by cytoskeleton proteins (**Figure S5F**). The protein modulator class includes chaperones and various kinases and G proteins regulators (**Figure S5C**). The cell signalling and the membrane/cell-cell contact classes are more complex and include many specialized proteins such as signalling molecules, receptors and transporters (**Figures S5B, E**).

Gene Ontology of Biological Processes (GO-BP) and Molecular Function (GO-MF) showed an enrichment in signal transduction and general biological processes across the entire time course (**Figures S6A, B**; **Supplementary Tables S4**, **S5**). However, several interesting dynamic functions were identified. For instance, transcription cofactors, transcriptional repressors and transcription factor binding were already enriched 1 minute after hormone exposure (**Figure S6B**) consistent with our previous observations of rapid transcription factor recruitment following hormone exposure (9, 19). We observed enrichment in ATP binding and Adenyl-ribonucleotide binding after 5 and 60 minutes of hormone exposure (**Figure S6B**), which may represent regulation of the two cycles of ATP dependent chromatin modifiers in response to progesterone (21, 57). KEGG pathway analysis reveals a significant enrichment in signalling and in many cancer pathways, including Prostate, Glioma, CML, lung, AML, endometrial and pancreatic cancer, as well as focal adhesion and tight junctions (**Figure S6C**). Annotated signalling cascades were significantly enriched at all time points in response to progestin, including MAPK, neurotrophin (TRK), ERBB, FC-receptor and insulin signalling (**Figure S6C**).

### Specific pathways: roles of AMPK, insulin TNFa, and PIK3

K Means clustering analysis revealed six patterns of regulation over the time course (**Figure 3C**). Similarity analysis of all phosphorylation sites within all clusters shows several interesting dynamics. First, “Early-risers” cluster 1 and 4, are positively correlated on the similarity matrix and show their initial increase in phosphorylation early at 1 and 5 minutes, respectively (**Figure 3D**). GO-BP analysis of the proteins contained within the “early riser” clusters shows an enrichment in signal regulation, and signalling cascades including Hippo, NFk-B and MAPK pathways (**Figure 3E**; **Supplementary Table S9**). Second, clusters 3 and 6 show an opposing nature (negative correlation **Figure 3D**). This antagonistic behavior of the two clusters is clearly shown averaging the signal of all phosphorylation within each cluster (**Figure 3F**). Corum (comprehensive resource of mammalian protein complexes) analysis of the significantly enriched protein complexes contained within clusters 3 and 6 (**Supplementary Table S6**) showed that most protein complexes were enriched in one cluster or the other (**Figure 3G**), likely representing crosstalk. Ten protein complexes were found to be enriched in both cluster 3 and 6, having phosphorylation sites within the same protein complex regulated in an opposite manner (**Figure 3G**).

One such complex was the cMyc-ATPase-Helicase complex, which contains 5 proteins; cMyc, the chromatin remodelling component BAF53, the ATP-dependent helicases RUVBL1 and RUVBL2 (also known as TIP48 and 49) and the histone acetyltransferase, TRRAP. This complex is involved in chromatin organization, histone acetylation and transcriptional regulation (58). Analysis of the phosphorylation sites showed that two sites (S373, T58) within Myc were increased early after hormone exposure, and decreased after 60 minutes, whereas one site of BAF53 (S233) shows the opposite dynamic (**Figure 3H**). Database analysis also reveals a strong overexpression of BAF53 in tumor versus normal samples in multiple cancer types (**Figure 3I**). Myc has an important role in breast cancer growth *via* the activation of AMPK (59).

The AMP-activated protein kinase (AMPK), exhibited a decrease in T183 phosphorylation in response to hormone. This site is phosphorylated by CAMKK1 or 2 (60). AMPK is a master sensor, and its activation inhibits several kinase pathways including mTOR, NfkB, JAK/STAT, insulin and Hippo (61–64). In addition, active AMPK inhibits the phosphorylation of PR S294, PR recruitment to chromatin and the activation of progesterone regulated genes (65). Activation of the kinase, specifically requires the phosphorylation of AMPK at T183 by CAMKKs, and de-phosphorylation of this site has been shown to be induced by estrogens and androgens in adipocytes (66, 67). Previous published results and the data presented here suggests a model where AMPK must be silenced in order for regulatory pathways described and PR itself to be active, which is what we observe within 1 minute of progesterone stimulation (**Figures 3J, K**).

**Figure 4:**
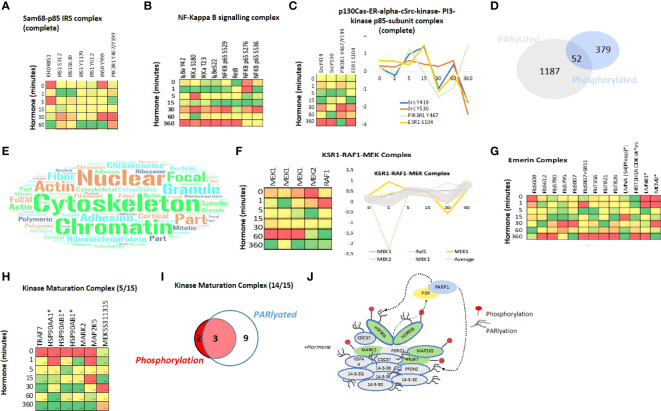
Protein Complex analysis and Overlap of PARylation and Phosphorylation in response to Progesterone. Heatmaps showing the phosphorylation of proteins within the Sam68-p85 IRS **(A)** and NF-kappa B **(B)** signalling complexes in response to hormone over time. **(C)** Heatmap showing the phosphorylation of proteins of the p130 Cas-ER-Src-PI3K complex in response to hormone over time, the coordinated phosphorylation of each phosphosite individually is represented as a line graph (right panel). **(D)** Venn diagram showing the overlap of proteins which contain either a phosphorylation site (379), PARylation site (1187) or both PTMs within the same protein after hormone exposure in T47D cells (52). **(E)** Word cloud representation showing the GO-cellular component enrichment analysis of the 52 proteins identified as phosphorylated and PARylated in response to hormone **(D)**. **(F)** Heatmap showing the phosphorylation of components of the KRS1-RAF1-MEK signalling complex in response to hormone over time, all proteins shown are phosphorylated and PARylated and the dynamics of individual sites is shown on the right panel. **(G)** Heatmap showing the phosphorylation proteins of the Emerin complex in response to hormone over time. **(H)** Heatmap showing the phosphorylation of proteins (5/15) within the Kinase Maturation complex in response to hormone over time, phosphorylation dynamics of individual sites is shown (lower panel). **(I)** Venn diagram showing the phosphorylation and or PARylation of proteins contained within the kinase maturation complex; 14/15 protein components of the complex contain at least one of the PTMs. **(J)** Schematic representation of the complex components, PARylated proteins are indicated by blue circle, Phosphorylated by red (right panel). *Indicates proteins which are also PARylated.

Further in-depth analysis of the complexes which are regulated by phosphorylation in response to progestin revealed a full list of complexes with at least 2 proteins phosphorylated in response to hormone. The Sam68-p85 P13K-IRS-1-IR signalling complex, which encompasses the insulin receptor (INSR), the insulin receptor substrate 1 (IRS1), the KH domain containing transduction-associated protein 1 (Sam68) and the phosphatidylinositol 3-kinase regulatory subunit alpha (GRB1). This protein complex is involved in insulin signalling and has been proposed to provide a link between the PI3K pathway and other signalling cascades of insulin or p21/RAS (68). We observed a dynamic phosphorylation of several sites within the complex (**Figure 4A**), including 4 distinct phosphorylation events within IRS1, two of which peak at 1 minute (S312, S639) and two sites where the peak in phosphorylation is observed at 60 minutes (Y1179, and Y612). S312 has been shown to be directly phosphorylated by c-Jun N-terminal kinase (JNK1) in breast cancer signalling (69) and this phosphorylation inhibits its interaction with IKKA. S639 is phosphorylated by mTOR has been linked to PI3K/Akt/mTOR signalling in breast cancer (70, 71) and effects the intracellular localization of IRS1 (72). Y1179 has been reported to be phosphorylated by IGF1R or INSR itself (73) and Y612 phosphorylation activates the interaction with PIK3R1 (74).

The phosphorylation of several components of the TNFa/NFkB signalling complex were also identified (**Figure 4B**). This complex is involved in I-kB kinase/NF-kB signalling in tumor progression. Indeed, complex components IKKα, RelB and p52 are associated with decreased cancer-specific survival in ERa-positive breast cancer (75). This may be linked to the cancer stem cell niche, which we showed recently was present in T47D cells grown in 3D cultures (24). NFkB regulates self-renewal in breast cancer stem cell (BCSC) models and deletion of IKKα in mammary-gland epithelial cells affects progestin-driven breast cancer (76, 77). Indeed, the upstream activator RANK ligand (RANKL) and hence the RANK pathway promotes mammary tumor formation, (78), (76). Another example is the P130Cas-ER-cSrc-PIK3 kinase complex (**Figure 4C**). Which has been shown to induce transcriptional changes in response to oestrogen and mammary proliferation in breast cancer. The authors showed that estradiol triggers the association of ERa, c-Src, the p85 subunit of PI 3-kinase (PI3K) and p130Cas in a macromolecular complex and activates the c-Src kinase leading to p130Cas-dependent Erk1/2 phosphorylation (79, 80). Given the similarity of the phosphorylation dynamics, peaking early at 5 and 15 minutes across Src, PIK3 and ESR1 within the complex induced by progestin (**Figure 4C** right panel) this may (similarly to the induction by oestrogen shown by others) present a novel ERa-ERK-cSrc activation mechanism in response to progestin in breast cancer cells.

### Crosstalk between progestin induced phosphorylation and PARylation

Recently, it has been shown that the majority of PARylation events on eukaryotic nuclear proteins take place on serine residues rather than acidic residues as previously accepted (81–86). Given the enrichment of serine in the phosphorylation dataset (**Figure 2D**) and the importance of both PTMs in progestin gene regulation (21), we investigated the overlap between PARylation sites and phosphorylation sites within protein complexes. We identified 52 proteins, which were both phosphorylated and PARylated in response to progestins in T47D breast cancer cells (**Figure 4D**). Cellular component analysis of this set of 52 proteins indicates a significant enrichment in nuclear, cytoskeleton and chromatin associated proteins (**Figure 4E**; **Supplementary Table S7**), in line with the well described role of PAR in the nucleus, nuclear organization, chromatin organization, relaxation and transcriptional regulation (87–90). This finding may indicate a crosstalk between PARylation and phosphorylation with regards to nuclear structure and chromatin organization. Analysis of complexes significantly enriched within this group of proteins revealed 12 protein complexes (**Supplementary Table S8**), which contained proteins both PARylated and phosphorylated. One of them is the KSR1-RAF1-MEK complex composed of MEK1 and 2, both PARylated and phosphorylated, and RAF1 which is phosphorylated (**Figure 4F**). This complex is involved in the MAKPKKK cascade, and in response to EGF it activates BRAF mediated phosphorylation of MEK1, at 3 sites, and MEK2, which activate MAPK1 and 3 (91). In our dataset we observe a clear change in phosphorylation of all members of the complex in response to progestin (**Figure 4F** right panel).

We also observed the phosphorylation and PARylation of the Emerin complex (**Figure 4G**). This complex is involved in DNA replication, transcription and structural integrity of the nucleus, specifically of the inner nuclear membrane (92). Depletion of Emerin results in changes in the organization and dynamics of the nucleus, increased chromatin mobility and a mis-localization of chromosome territories (93). Within this complex we find proteins phosphorylated, PARylated, or phosphorylated and PARylated (**Figure 4G**). Given the role of PAR in the structure of the nucleus, this complex may present an interesting example for studying the PARylation, phosphorylation crosstalk.

As discussed, prior to hormone exposure PR is present in an inactive complex with the HSP70 and 90 proteins as part of the Kinase Maturation Complex. We know that progestins promote the phosphorylation and dimerization of the receptor and we found that phosphorylation of the HSP90 and 70, along with other members of the complex, is initiated within 1 minute of hormone exposure (**Figure 4H**), again showing a rapid and concerted phosphorylation of several members of the complex (**Figure 4H**). In addition, the HSPs are also PARylated as compared to other components where only phosphorylation (MARK2, MAP2K5) or PARylation (14-3-3 components) are present (**Figures 4I, J**). Further investigation regarding the crosstalk between PARylation and phosphorylation within protein complexes will be the focus of future studies.

### Progesterone signalling network generation

In order to understand the crosstalk between the signalling pathways activated by Progesterone in breast cancer cells, a Protein-Protein Interaction (PPI) network was generated using all identified phosphorylation sites (**Supplementary File: Network session 2**), based on known PPI (evidence based). The resulting network consists of 427 nodes (proteins) and 4309 unique interactions (edges) (**Figure S7A**). Pathway analysis was carried out on this network, using Genemania™, and 23 statistically significant (p<0.01) pathways were identified (**Supplementary Tables S9** and **S10**). The proteins and interactions (nodes and edges) associated with each pathway were selected and new networks generated (**Supplementary File: Network session 2**). Several of which are shown in **Figures S7B–G** and discussed briefly below.

One such pathway, the Fc receptor signalling pathway was enriched (**Figure S6C**) and the PPI network is shown in **Figure S7B**. Fc receptors are cell surface proteins that recognize the FC fragment of antibodies, mainly on immune cells. However, recent studies have shown that different subsets of Fc receptors may play a role in tumor cells (94). In particular, it was shown that T47D cells express the FcyRI (CD64). These FC-receptor expressing breast cancer cells can activate the tyrosine kinase signal transduction pathway. Indeed, T47D cells treated with selective tyrosine kinase inhibitors do not proliferate in a FC receptor- tyrosine kinase signalling dependent manner (94).

As mentioned before (**Figure S6C**), another pathway identified as activated in response to progestin is the ERBB-EGF network (**Figures S7D, H**). ERBB2 (HER2) is overexpressed in 15-20% of breast cancer in response to EGF activation, and plays a major role in EMT (95). PR interacts with ERBBs and induces the translocation of ERBB2-PR-STAT3 complex to the nucleus. ERBB2 acts as a co-activator of STAT3 and drives the activation of progestin regulated genes, especially genes such as Cyclin D1 that do not contain HREs (96, 97). Blocking PR signalling in PR-ERBB2 positive breast cancer patients has been suggested as a treatment (98). The ERBB pathway may represent a new mechanism for further study to understand the activation of these “non-classical” PR dependent genes in response to progestin. In addition, to the role of ERBB2, the role of ER activation in response to progesterone in breast cancer cells is also critical, as shown in **Figure 4C** we observe the coordinated activation of the ESR1-Src-PIK3 complex peaking at 15 minutes following hormone exposure. The phosphorylation site of ESR1 which increases is S104. ER S104 phosphorylation is essential for ER activity (99) and it has been suggested that hyperphosphorylation of ER at these sites may contribute to resistance to tamoxifen in hormone receptor positive breast cancer (100–102). ER S104 phosphorylation by ERK has been shown previously in response to oestrogen and EGF but not progesterone exposure. In addition, ER S104 has been implicated in mTOR signalling (103). Given the phosphorylation of ER, the dynamic activation of the ER membrane complex and the role of mTOR in AMPK and insulin signalling described earlier (**Figures 3J** and **4C**) this phosphorylation site may present a key step in the cellular response to progesterone in breast cancer.

A pathway exhibiting strong activation by progestins is the Insulin signalling (**Figure S6C**; **Figure S7F**). Insulin-like growth factors (IGFs) and progestins both play a major role in normal mammary gland development and R5020 has been shown to induce the expression of insulin receptor substrate-2 in MCF7 cells (104, 105). Moreover, IGF signalling *via* IRS2 is known to be essential for breast cancer cell migration. It has also been shown that R5020 pretreatment followed by IGF stimulation increases binding of IRS to PI3K-p85 regulatory complex, which in turn activates ERK and AKT signalling (106). Interestingly, not only do we observe the activation of the insulin pathway in network analysis (**Figure S7F**, but also the coordinated phosphorylation of all members of the IRS-PIK3 complex (**Figure 4A**), indicating that indeed progesterone stimulation of T47D cells activates not only the insulin pathway in general terms but also triggers the coordinated regulation of complexes within it.

## Discussion

The data presented here provides a source of knowledge for the scientific community with regards to progesterone induced gene expression, and the signalling pathways involved. We have shown the rapid induction of phosphorylation using two distinct technologies (**Figures 1**, **2**). Pathway analysis showed a strong enrichment in pathways associated with cancer, known and novel Pg-dependent signalling events (**Figure 3A**). But also identified signalling pathways not previously known to mediate progesterone action in breast cancer cells, such as MNK1/EIF4E pathway and the connection between CDK2, Cdc25 and the MAPK pathway.

Cellular component analysis confirmed our expectations and the statistically significant activation of the cell membrane within 1 minute of hormone exposure (**Figure 3B**), but also revealed a consistent (over all members) phosphorylation peak within proteins associated within the mitochondria at 15 minutes after hormone exposure (**Figures 3B** and **S4E**). Mitochondrial activation in response to progesterone in breast cancer cells has not been extensively studied yet. Indeed, ATP synthesis 45-60 minutes after hormone stimulation is independent of mitochondrial involvement (21). However, there are some interesting findings in the literature. Following the observation that the PR negative cell line MCF10A exhibits a progestin-induced cell proliferation (107). Behera and colleagues showed that MCF10A responded to R5020 with an increase in mitochondrial activation (108). Given the absence of the nuclear PR in these cells they hypothesized that the activation of progestin-induced cell growth was due to non-genomic metabolic effects, mediated by a yet undiscovered receptor. We propose that the observed mitochondrial activation in T47D in response to progestin (**Figures 3B** and **S4E**) suggests the existence of a third and interconnected hormonal signal transduction pathway *via* the mitochondria (109, 110).

Dynamic phosphorylation analysis over time reveals distinct groups or clusters of phosphorylation events which follow a similar time response; such as early risers, sustained or late (**Figure 3C**). Similarity analysis of these dynamic phosphorylation sites reveals some interesting crosstalk between protein complexes not previously identified as players in progesterone signalling (**Supplementary Table S6**), and complexes where a mobilization of phosphorylation (showing similar dynamics) was observed within the whole macromolecular complex; such as PIK3, NFkB (**Figures 4B, C**).

Overlap of phosphorylation sites with existing PARylation, revealed 52 proteins for which both phosphorylation and PARylation was found. The data also clearly shows an enrichment in protein complexes that play a role in the structural organization of the nucleus (**Figures 4D, E**), specifically the Emerin complex and Lamin (**Figure 4G**). The key location of these complexes at the nuclear membrane suggests that perhaps these two PTMs may affect and play a role in the dynamic structure of the nucleus. This could be explored in the future by global chromatin proximity Hi-C experiments. The complexes identified in this study and the dual post translational modification of proteins with known important roles within the cell may provide exciting opportunities for future studies which aim to understand the crosstalk between Serine PARylation and phosphorylation in the context of nuclear architecture, signalling and breast cancer progression (**Supplementary Table S7**).

**Figure 5:**
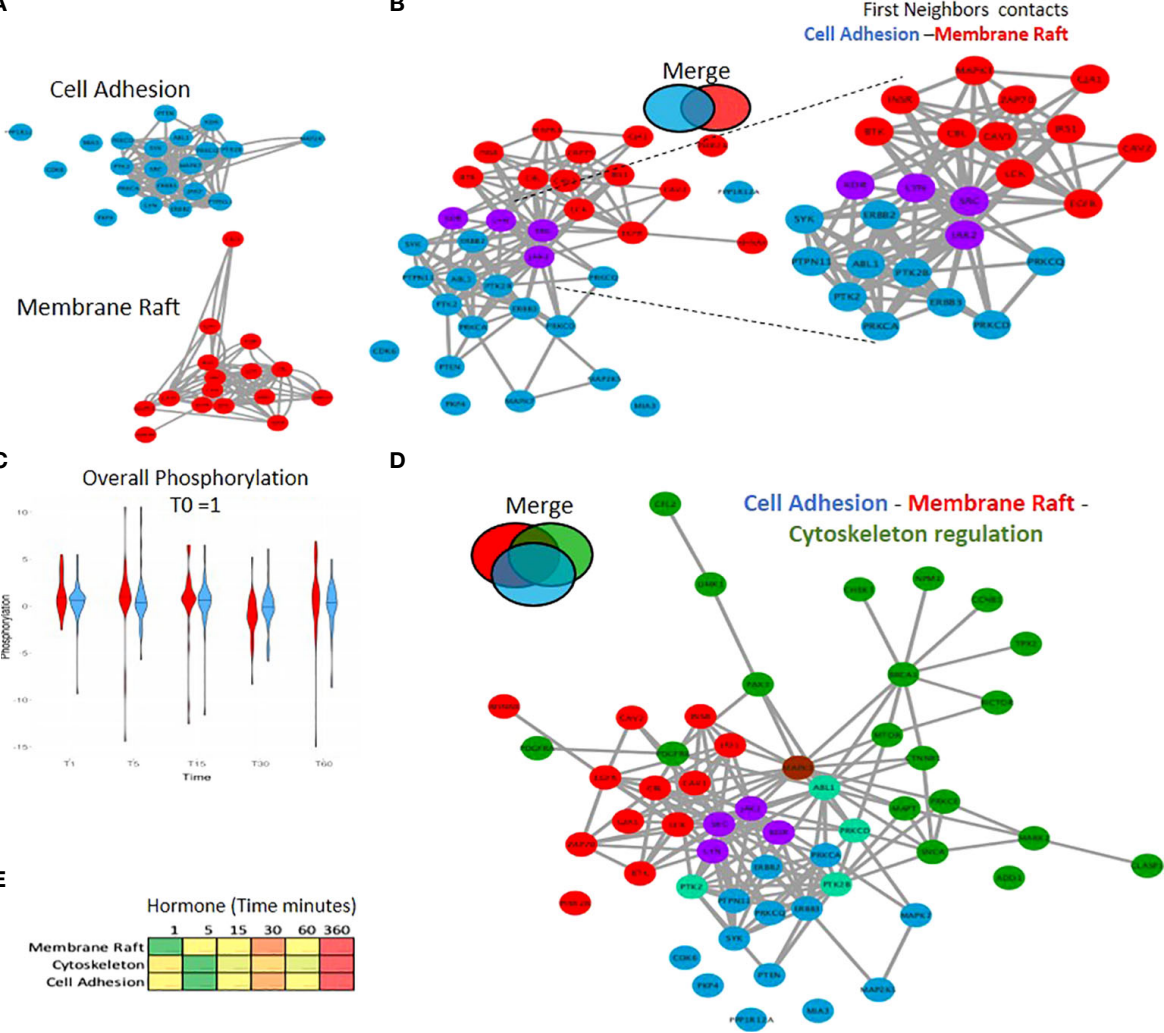
Combining PPI networks from distinct cellular compartments reveals a coordinated crosstalk. **(A)** PPI network showing the significantly regulated phosphorylated proteins located in cell adhesion (blue) and the membrane raft (red) identified in response to hormone. **(B)** Merge of the cell adhesion network **(A)** blue and membrane raft network **(A)** red. The two networks connect based on known PPI however no protein was identified as annotated in both sets. This integration of the two networks is highlighted (right panel) where proteins from each network were selected based on having a first neighbor with a protein of the other network. **(C)** Violin plot showing the average phosphorylation of proteins over time in response to hormone within the membrane raft or cell adhesion networks. Data is normalised to time 0 = 1. **(D)** Merge of Cell Adhesion-Membrane **(B)** and the cytoskeleton networks. The two networks are merged based on known PPI. Proteins annotated in more than one function are colored based on the Venn diagram (i.e. cytoskeleton and cell Adhesion; light green, membrane raft and cytoskeleton; brown). **(E)** Heatmap showing the average phosphorylation of all proteins within each network in response to hormone over time, showing the activation of the membrane raft first at 1 minute followed by the cytoskeleton and cell adhesion.

In addition to pathway analysis at the single network level (**Figure S7**), wherein we identified pathways such as insulin, Fc-receptor and ERBB signalling, it is also clearly important to consider the connection between pathways and networks as a whole. One such example is the connections between the phosphorylation events within the cytoskeleton, membrane raft and proteins associated with cell adhesion. We observe phosphorylation events in multiple proteins within both cell adhesion and the membrane raft, forming tight strongly connected PPI networks (**Figure 5A**). A network merge of these two pathways reveals 4 key proteins which are present in both (JAK2, SRC1, LYN and KDR), indicating a strongly connected network (**Figure 5B**), which in addition to common members exhibits a large first neighbor selection between the two initial pathways (selection of only direct PPI) (**Figure 5B** right panel) with a similar phospho kinetic pattern (**Figure 5C**). Incorporation of the significantly enriched phosphorylated proteins within the cytoskeleton (**Figure 3B**; **Figure S6D**; **Supplementary Table S3**) into the merged network (**Figure 5B**) results in a larger global connected network (**Figure 5D**) which supports the activation within the membrane proteins after 1-minute following hormone exposure that triggers the subsequent cascades of phosphorylation in the cytoskeleton (**Figure 5E**). These findings clearly show the importance of studying the pathways not in isolation, but rather in connection with each other.

**Figure 6:**
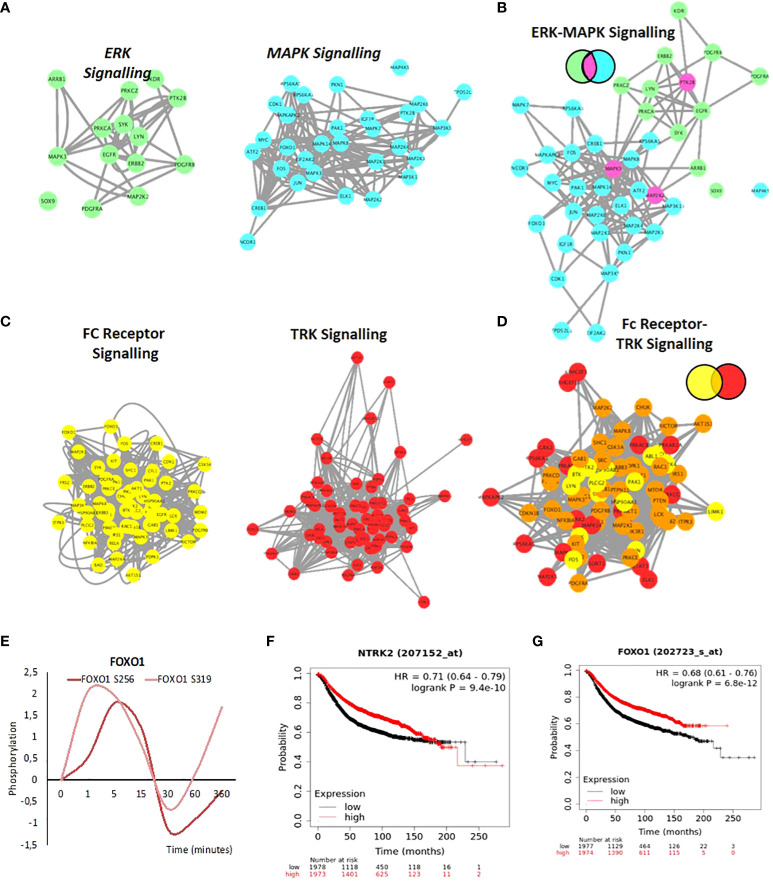
Network Integration of signalling networks identified in response to hormone. **(A)** PPI network showing the phosphorylated proteins present within the ERK signalling cascade (green) and the MAPK cascade (blue) identified in response to hormone. **(B)** Merge of ERK-MAPK networks **(A)**. The two networks are merged based on known PPI. Proteins annotated in both pathways are colored based on the Venn diagram (fuchsia). **(C)** PPI network showing the phosphorylated proteins present within the FC-receptor (yellow) and TRK-neurotrophin (red) signalling pathways (yellow) identified in response to hormone (left and middle panel). **(D)** Merge of FC-receptor and TRK neurotrophin networks. The two networks are merged based on known PPI. Proteins annotated in both pathways are colored based on the Venn diagram (orange). **(E)** Rapid and coordinated phosphorylation of FOXO1 S256 and FOXO S319 in response to hormone. Kaplan Meyer overall survival of patients stratified based on the expression of NTRK2 **(F)** and FOXO1 **(G)** in breast cancer patients (p=9.4E-10 and 6.8e-12 respectively). All networks, PPIs and integrated cascades are supplied in Cytoscape session 2.

Other examples of connectivity are observed between the ERK subgroup and the MAPK cascades and the FC Receptor and TRK signalling. ERK and MAPK form a strong network (**Figures 6A, B**). As discussed, earlier Fc-receptor signalling shows a strong activation (**Figure S7B**). The tropomyosin receptor tyrosine kinases (TRKs) are primarily known for their roles in neuronal differentiation and survival. However, increasing evidence shows that TRK receptors can be found in a host of mammalian cell types to drive several cellular responses (111, 112). Aberrations in TRK signalling, which can occur through events such as protein overexpression, alternative splicing, or gene amplification, can lead to disease such as cancer (113–115). The receptor tyrosine kinase NTRK2, activates GRB2-Ras-MAPK cascade in neurons and increases secretion by epithelial cells in culture in response to oestrogen or progestin treatment and NTRK2 was identified as differentially expressed between stromal and epithelial breast cells which may have implications in invasion and metastasis (116). Merging of Fc-receptor and TRK signalling pathways (**Figure 6C**) shows a strong protein overlap and a dense connected network with FOXO1 at the center (**Figure 6D**). FOXO1 is phosphorylated after 1 to 5 minutes of progestin exposure (**Figure 6E**). Phosphorylation of FOXO1 by PKB/Akt has been shown to be important for the binding to 14-3-3 proteins on chromatin (117–120). The role of these two pathways in progesterone induced gene regulation has not been shown previously. FOXO factors have a key role to play in tumor resistance to therapy and patient outcome (121). Interestingly, from a clinical perspective, stratifying patients based on either the expression levels of NTRK2 (TRKB) or FOXO1 is predictive of a good prognosis (overall survival) in breast cancer, similar to prognosis based on PR expression (**Figures 6F, G**).

The examples described here in addition to other examples contained within the data for future discovery show the importance of network connectivity in trying to understand not only individual proteins or pathways but the significant overlap between the pathways within the signalling network activated by progesterone in breast cancer. Further analysis of the detected connections and identification of the key regulators may provide a source of targets for drug discovery aiming at the treatment of hormone receptor positive breast cancer patients.

## Conclusions

The data presented here, reveals a high level of complexity in progesterone signalling in T47D breast cancer cells, shedding new light on known proteins and signalling pathways. Functional analysis reveals the activation of known pathways such as MAPK cascade but also the activation of signalling cascades not previously associated with progesterone signalling such as TRK, TNFa and ERBB. Our analysis indicates that there is a full cellular coordinated response, with proteins activated in different cellular compartments at different times following hormone exposure, in addition to the activation of whole protein complexes previously not associated with progesterone signalling. One limitation of our study is the use of a single breast cancer cell line, future work will aim to address this limitation with the use of additional cell lines. However, we believe that this signalling network and the phosphosites identified represent a rich resource for the breast cancer research community, opening up new lines of research and ideas for possible drug discovery projects for the benefit of breast cancer patients.

## Data availability statement

‘The original contributions presented in the study are included in the article/**Supplementary Material**. Further inquiries can be directed to the corresponding authors.

## Authors contributions

Experimental design, RW and MB. Bioinformatic analysis, JO, JC-C, and RW. Manuscript writing and editing, RW and MB. Experiments, RW. All authors contributed to the article and approved the submitted version.

## Funding

This research was supported by European Research Council (Project “4D Genome” 609989), the Ministerio de Economía y Competitividad (Project G62426937) and the Generalitat de Catalunya (Project AGAUR SGR 575 and AGAUR 2019PROD00115/IU68-016733), European Research Council -Proof Of Concept (Project “Impacct” 825176).

## Acknowledgments

We acknowledge the support of all members of the Chromatin, Gene Regulation laboratory and members of the Gene Regulation Cancer and Stem Cells department at Centre for Genomic Regulation (CRG, Barcelona Spain) and the CRG/UPF proteomics unit.

## Conflict of interest

The authors declare that the research was conducted in the absence of any commercial or financial relationships that could be construed as a potential conflict of interest.

## Publisher’s note

All claims expressed in this article are solely those of the authors and do not necessarily represent those of their affiliated organizations, or those of the publisher, the editors and the reviewers. Any product that may be evaluated in this article, or claim that may be made by its manufacturer, is not guaranteed or endorsed by the publisher.

## Supplementary material

Supplementary Figure 1 Prior Knowledge Network (PKN) Progesterone Signalling. **(A)** Edge directed PKN network was manually curated from the literature. Annotated phosphorylation events, interactions, dissociations and cellular compartment are indicated. Network is available as a cytoscape network session (cys) or interaction (sys) file containing references for all edges present as shown in **(B)** (See **Supplementary File Network 1**).

Supplementary Figure 2 Antibody Array controls and data analysis. **(A)** Schematic indicating the experimental procedure, quality control checks, and filtering applied to the antibody array experiments. **(B)** Number of phosphosites identified per protein. Tau, PTK2, RPS6KA1 and RB1 are highlighted as they have multiple sites identified. **(C)** Functional classification of the proteins identified as significantly phosphorylated at each time point **(D)** KEGG pathway analysis, showing significant pathways (-log_10_ p-value) at each time point. **(E)** Heatmap representation of GO biological process data, showing significant (-log_10_ p-value) processes at each time point. **(F)** Heatmap representation of GO molecular function data, showing significant (-log_10_ p-value) functions at each time point.

Supplementary Figure 3 Phosphoproteomic data acquisition and controls. **(A)** Correlation of triplicate samples from each of the time points. **(B)** Number of phosphosites identified per protein, the proteins showing multiple sites per protein are highlighted. **(C)** KEGG pathway analysis, showing significant pathways (-log_10_ p-value) at each time point. **(D)** Heatmap representation of GO-biological process data, showing significant (-log_10_ p-value) processes at each time point. **(E)** Heatmap representation of GO-molecular function data, showing significant (-log_10_ p-value) functions per time point.

Supplementary Figure 4 Combining Antibody Array and Phosphoproteomic LC-MS-MS datasets. **(A)** Schematic representation showing the methodology and overlap combining antibody array and LC-MS-MS datasets. **(B)** PCA analysis of phosphorylation datasets. **(C)** Number of phosphosites identified per protein, the names of proteins showing multiple sites per protein are highlighted. **(D)** Venn diagram showing the overlap of phosphosites per time point. **(E)** Up and down regulated phosphorylation sites identified per time point. **(F)** Phosphorylation levels of the proteins identified as significantly regulated after hormone located within the mitochondria. **(G)** Analysis of the number of functions to which each unique protein was assigned **(H)** Venn diagram showing the overlap of protein functional class; Enzymes, Structural protein, Membrane-cell-cell contact, protein modulators and proteins with nucleic acid binding capacities.

Supplementary Figure 5 Functional Analysis of Proteins Identified. All proteins were assigned one or more function based on the GSEA database. Both Parent (outside/title), and children (within) are shown for each class and the proteins identified within that sub-group are shown. Nucleic acid binding **(A)**, Membrane/Cell-cell contact **(B)**, Protein Modulators **(C)**, Enzymes **(D)**, Cell signalling **(E)**, and Structural proteins **(F)**.

Supplementary Figure 6 Gene Ontology and Pathway analysis of combined dataset. **(A)** Heatmap representation of GO biological process data, showing significant (-log_10_ p-value) biological processes enrichment based on the protein phosphorylation at each time point. **(B)** Heatmap representation of GO molecular function enrichment, showing significant (-log_10_ p-value) functions at each time point following hormone. **(C)** KEGG pathway analysis, showing significant pathways (-log_10_ p-value) enriched at each time point following hormone exposure. **(D)** Protein protein interaction (PPI) network generated using proteins identified as phosphorylated following hormone and were assigned as cytoskeleton located.

Supplementary Figure 7 Pathway Network Generation in Breast Cancer cells in response to Hormone. **(A)** Protein protein interaction (PPI) network was generated using a full phosphorylation dataset encompassing 321 proteins (**Supplementary Material Network session 2**) in Cytoscape using Genemania™ only considering protein-protein interactions with experimental evidence (Supp. Materials and methods), each node represents and individual protein and interactions are represented by edges. Functional analysis was carried out to identify key pathways enriched within the full network (Full list **Supplementary Table 10**). Individual networks were generated from each function individually and are available within additional Network session 2. Graphs of several pathways determined to be enriched within the dataset are shown **(B)** Fc receptor, **(C)** MAPK, **(D)** EGF **(E)** ERK **(F)** Insulin, **(G)** TRK signalling, **(H)** ERBB.

Supplementary Table 1 Uniprot IDs of phosphorylated proteins identified in response to hormone. Time after hormone (minutes), data is normalised 0-1 row maximum and minimum.

Supplementary Table 2 KEGG pathway enrichment; the pathway term, p value and the proteins associated with the pathway are shown.

Supplementary Table 3 Cellular component enrichment analysis of phosphorylated proteins. The time after hormone in which they peak, the adjusted p value, and proteins associated with each specific cellular component are given.

Supplementary Table 4 Gene Ontology Biological Process enrichment analysis of phosphorylated proteins. The cluster in which the term is enriched, the adjusted p value, and proteins associated with each specific biological process are given.

Supplementary Table 5 Gene Ontology Molecular Function enrichment analysis of phosphorylated proteins. The cluster in which the term is enriched, the adjusted p value, and proteins associated with each specific molecular function are given.

Supplementary Table 6 Corum enrichment analysis of phosphorylated proteins. The p-value, and proteins associated with each complex are given.

Supplementary Table 7 Cellular component enrichment analysis of phosphorylated and PARylated proteins. The adjusted p value, and proteins associated with each specific cellular component are given.

Supplementary Table 8 Corum enrichment analysis of phosphorylated and PARylated proteins. The p-value, and proteins associated with each complex are given, phosphorylated proteins are highlighted in yellow.

Supplementary Table 9 Genemania analysis of phosphorylated proteins, all protein IDs are listed along with the GO: IDs for which they are associated.

Supplementary Table 10 Genemania analysis of phosphorylated proteins, the pathways enriched in Network 2 are shown. The q value and the number of occurrences in the network versus the occurrences in the Network are shown.

Network Session 1 Edge directed PKN network was manually curated from the literature. Annotated phosphorylation events, interactions, dissociations and cellular compartment are indicated.

Network Session 2 Protein interaction (PPI) network was generated using a full phosphorylation dataset encompassing 321 proteins in Cytoscape using Genemania™ only considering protein-protein interactions with experimental evidence each node represents and individual protein and interactions are represented by edges. Functional analysis was carried out to identify key pathways enriched within the full network. Individual networks were generated from each function individually and are available as unique networks within the Network session.

## Abbreviations

AKT, AKT serine/threonine kinase 1; ARTD1, ADP-ribosyltransferase diphtheria toxin-like 1; ATP, Adenosine triphosphate; BAF, BRG1-associated factor 53A; BCA, Bicinchoninic acid; CAMKK1, Calcium/calmodulin-dependent protein kinase kinase 1; CDK1/2, Cyclin dependent kinase; EMT, Epithelial to mesenchyme transition; ER, Oestrogen/Estrogen receptor; ERBB, Receptor tyrosine-protein kinase erbB-2; ERK1, Extracellular signal-regulated kinase 1; KH, K homology; MNK1, MAP kinase signal-integrated kinase 1; MSK, Mitogen and stress activated protein kinase 1; NUDT5, Nucleoside diphosphate-linked moiety X motif 5; NURF, Nucleosome-remodelling factor subunit; PAR, Poly-ADP-ribose; PARG, Poly-ADP-ribose glycohydrolase; PARP1, Poly-ADP-ribose polymerase; PCA, Principal component analysis; PGR, Progesterone Receptor; PKN, Prior knowledge network; PML, Prommyelocytic leukemia; PPI, Protein protein interaction; PRE, Progesterone responsive element.
