# Supplementary material for: Global signalling network analysis of luminal T47D breast cancer cells in response to progesterone
Source: Front Endocrinol (Lausanne). 2022 Aug 11;13:888802. doi: 10.3389/fendo.2022.888802 (PMC9403329; doi:10.3389/fendo.2022.888802)
Supplement: Supplementary Figure 1 — Prior Knowledge Network (PKN) Progesterone Signalling. (A) Edge directed PKN network was manually curated from the literature. Annotated phosphorylation events, interactions, dissociations and cellular compartment are indicated. Network is available as a cytoscape network session (cys) or interaction (sys) file containing references for all edges present as shown in (B) (See Supplementary File Network 1). [file DataSheet_1.pdf]

**Fig. S1**

**A**

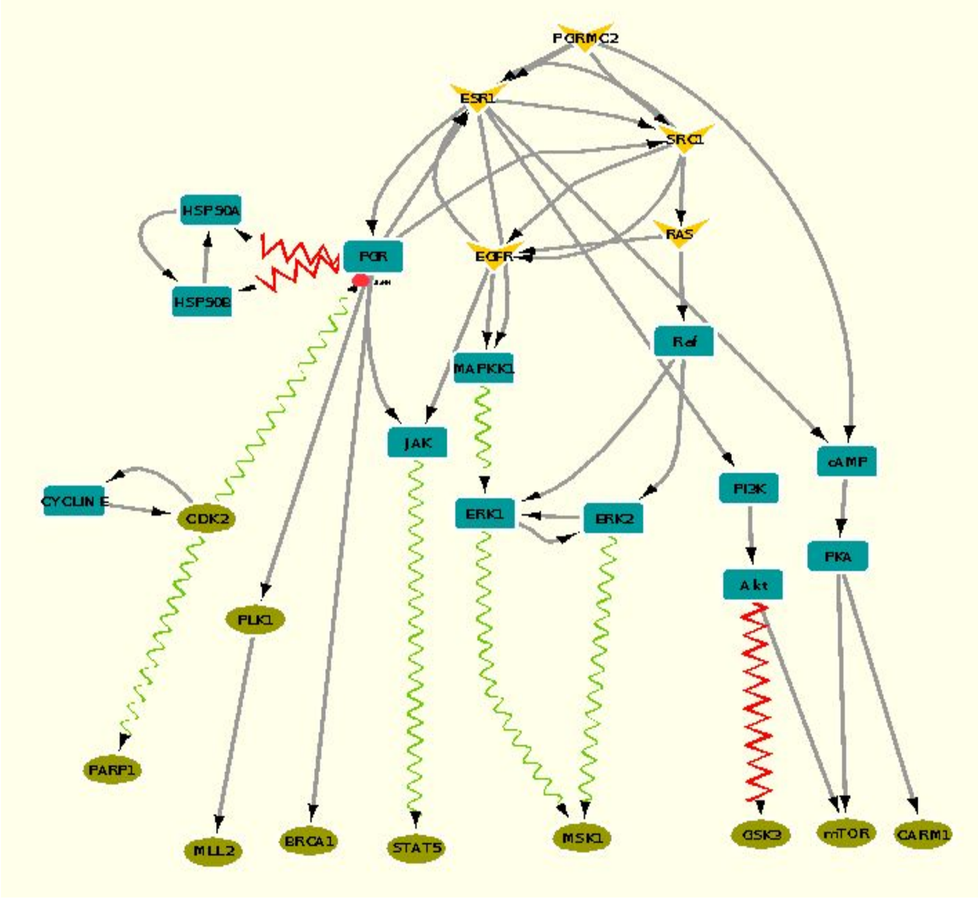


**B**

Progesterone literature

0 – 0 0 – 0

Table Panel

$f(x)$

| shared name | shared interaction | name | interaction | |
|---|---|---|---|---|
| CDK2 (interacts with) PGR | phosphorylates | CDK2 (interacts with) PGR | phosphorylates | Peirson 2004 Mullany |
| AKT (interacts with) GSK3 | dissociates from | AKT (interacts with) GSK3 | leaves | |
| AKT (interacts with) mTOR | interacts with | AKT (interacts with) mTOR | interacts with | |
| CDK2 (interacts with) CYCLIN E | interacts with | CDK2 (interacts with) CYCLIN E | interacts with | |
| CDK2 (interacts with) PARP1 | phosphorylates | CDK2 (interacts with) PARP1 | phosphorylates | |
| CYCLIN E (interacts with) CDK2 | interacts with | CYCLIN E (interacts with) CDK2 | interacts with | |
| EGFR (interacts with) ESR1 | interacts with | EGFR (interacts with) ESR1 | interacts with | |
| EGFR (interacts with) Jak2 | interacts with | EGFR (interacts with) Jak2 | interacts with | |
| EGFR (interacts with) MAPKK1 | interacts with | EGFR (interacts with) MAPKK1 | interacts with | |
| EGFR (interacts with) MEK | interacts with | EGFR (interacts with) MEK | interacts with | |
| ERK1 (interacts with) ERK2 | interacts with | ERK1 (interacts with) ERK2 | interacts with | |
| ERK1 (interacts with) MSK1 | phosphorylates | ERK1 (interacts with) MSK1 | phosphorylates | |
| ERK1 (interacts with) PGR | phosphorylates | ERK1 (interacts with) PGR | phosphorylates | |
| ERK2 (interacts with) ERK1 | interacts with | ERK2 (interacts with) ERK1 | interacts with | |
| ERK2 (interacts with) MSK1 | phosphorylates | ERK2 (interacts with) MSK1 | phosphorylates | |
| ESR1 (interacts with) MAPKK1 | interacts with | ESR1 (interacts with) MAPKK1 | interacts with | |
| [illegible] | [illegible] | [illegible] | [illegible] | |

**Fig. S2**

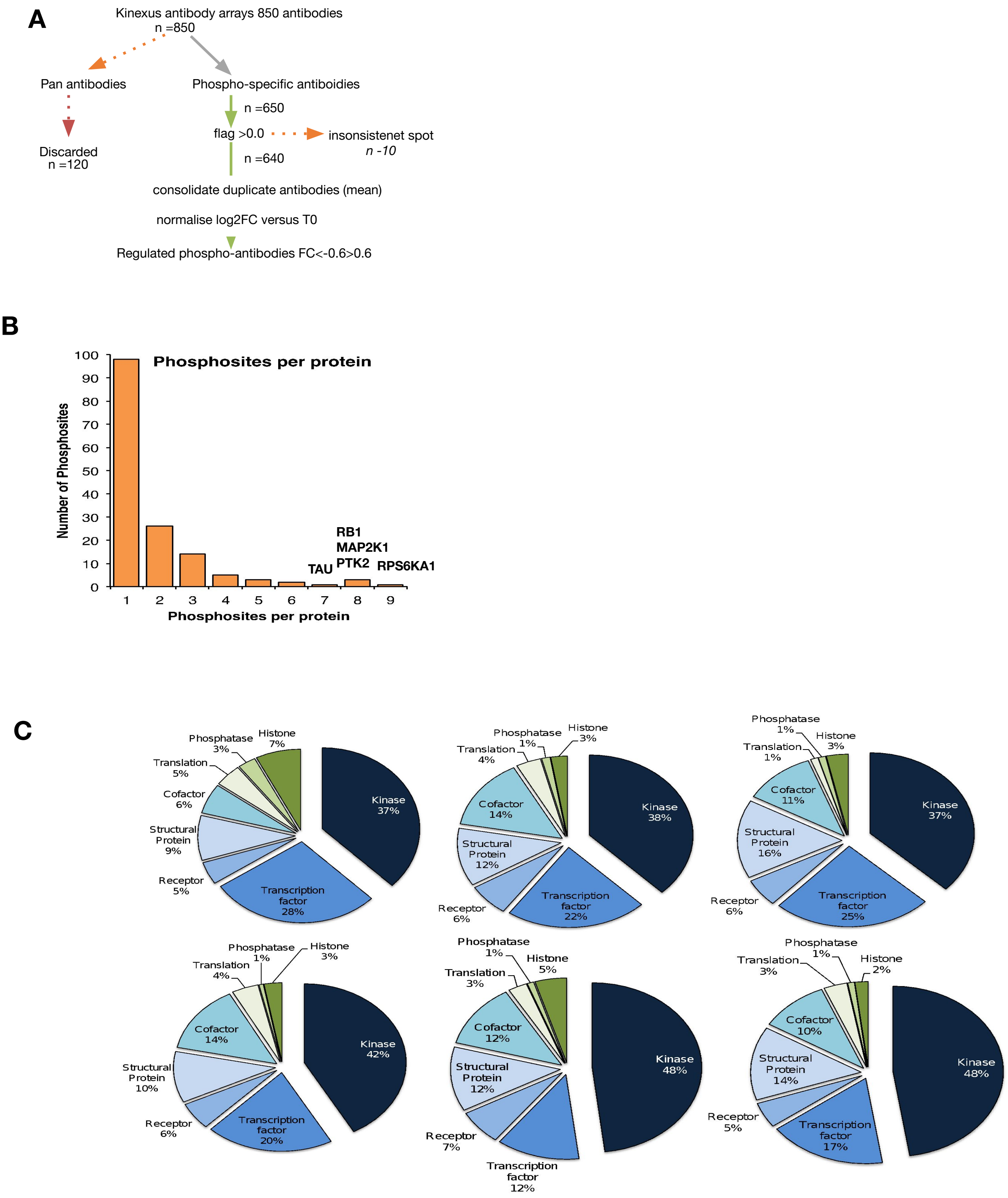
A
Kinexus antibody arrays 850 antibodies
n =850
Pan antibodies
Phospho-specific antiboidies
n =650
Discarded
n =120
flag >0.0
insonsistenet spot
n -10
n =640
consolidate duplicate antibodies (mean)
normalise log2FC versus T0
Regulated phospho-antibodies FC<-0.6>0.6
B
Phosphosites per protein
Number of Phosphosites
0
10
20
30
40
50
60
70
80
90
100
1
2
3
4
5
6
7
8
9
Phosphosites per protein
TAU
RB1
MAP2K1
PTK2
RPS6KA1
C
Phosphatase 3%
Histone 7%
Translation 5%
Cofactor 6%
Structural Protein 9%
Receptor 5%
Kinase 37%
Transcription factor 28%
Phosphatase 1%
Histone 3%
Translation 4%
Cofactor 14%
Structural Protein 12%
Receptor 6%
Kinase 38%
Transcription factor 22%
Phosphatase 1%
Histone 3%
Translation 1%
Cofactor 11%
Structural Protein 16%
Receptor 6%
Kinase 37%
Transcription factor 25%
Phosphatase 1%
Histone 3%
Translation 4%
Cofactor 14%
Structural Protein 10%
Receptor 6%
Kinase 42%
Transcription factor 20%
Phosphatase 1%
Histone 5%
Translation 3%
Cofactor 12%
Structural Protein 12%
Receptor 7%
Kinase 48%
Transcription factor 12%
Phosphatase 1%
Histone 2%
Translation 3%
Cofactor 10%
Structural Protein 14%
Receptor 5%
Kinase 48%
Transcription factor 17%

**Fig. S2 continued**

D

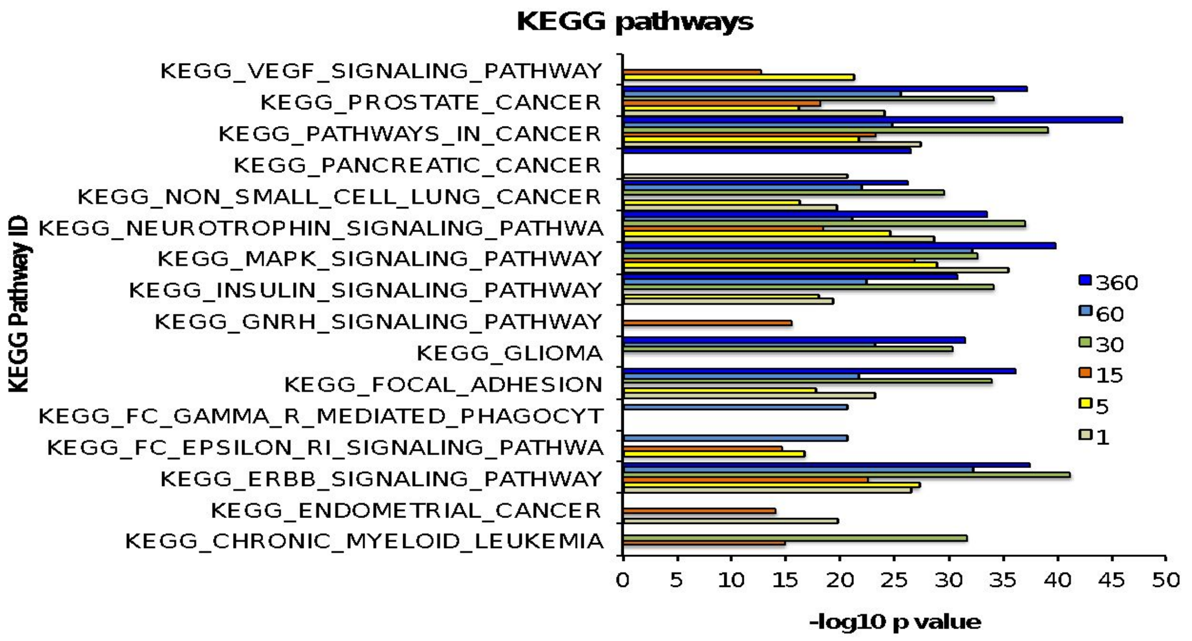


E

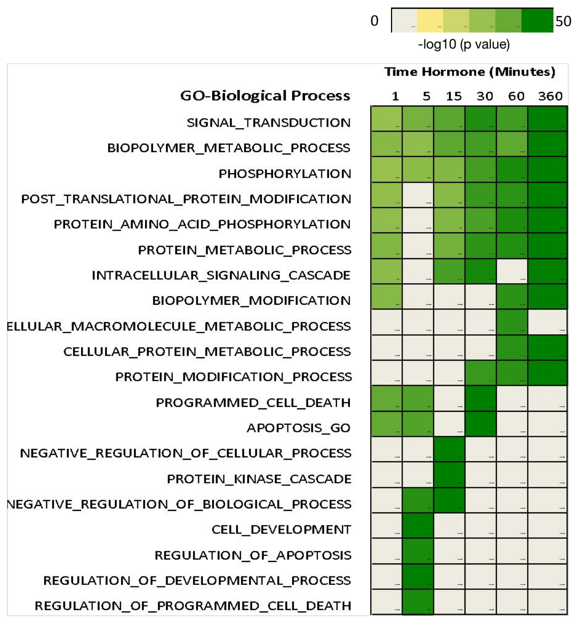


F

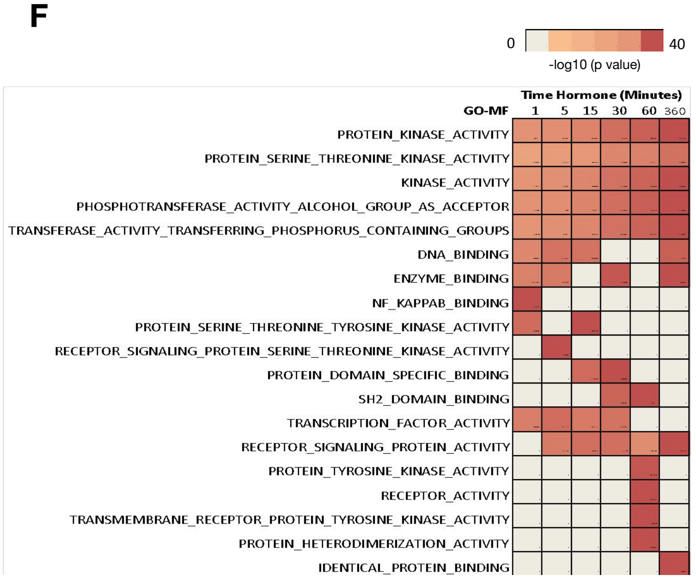

**Fig. S3**

A

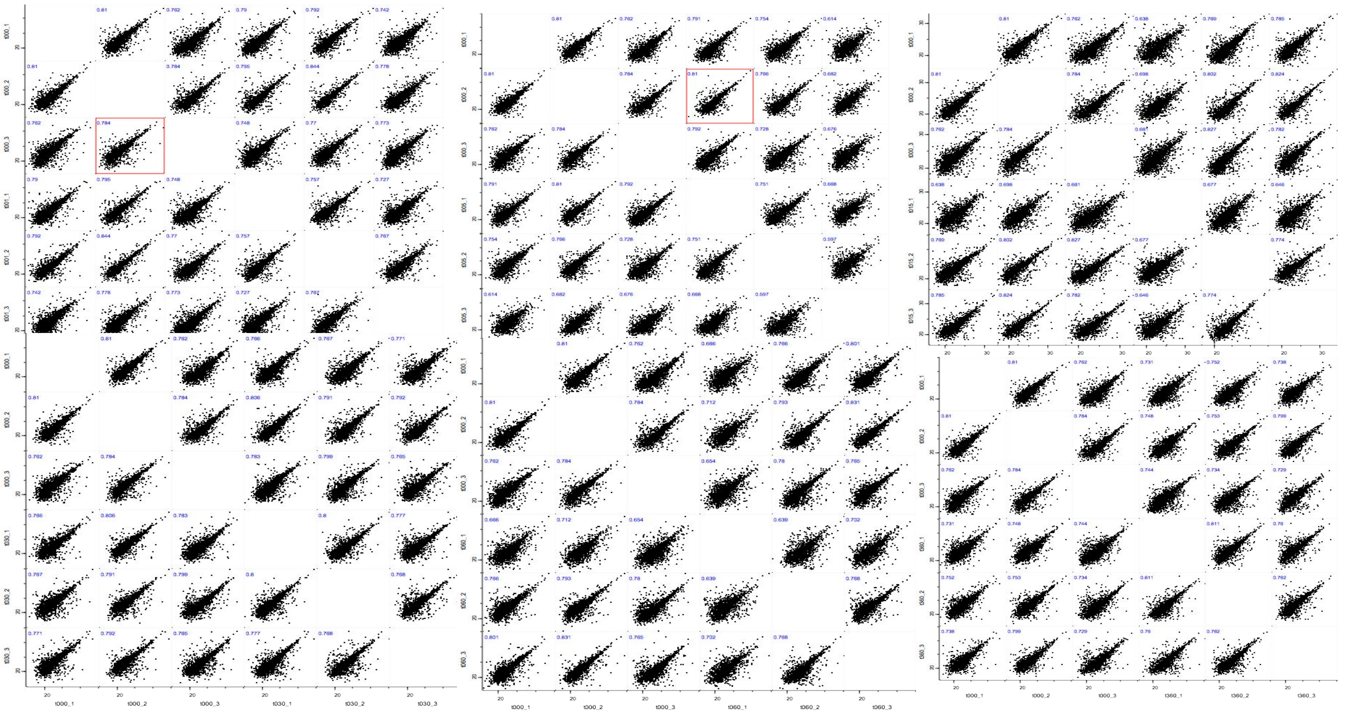

B

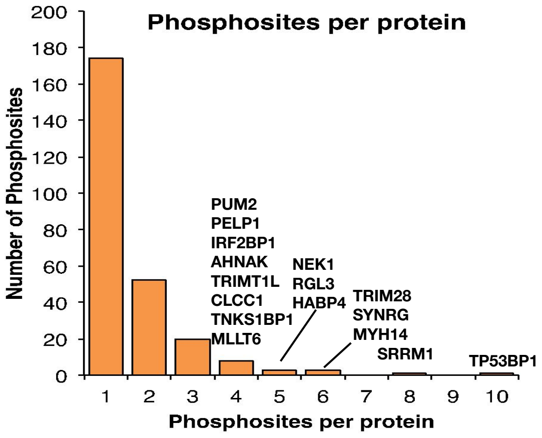
Phosphosites per protein
Number of Phosphosites
Phosphosites per protein
PUM2
PELP1
IRF2BP1
AHNAK
TRIMT1L
CLCC1
TNKS1BP1
MLLT6
NEK1
RGL3
HABP4
TRIM28
SYNRG
MYH14
SRRM1
TP53BP1

C

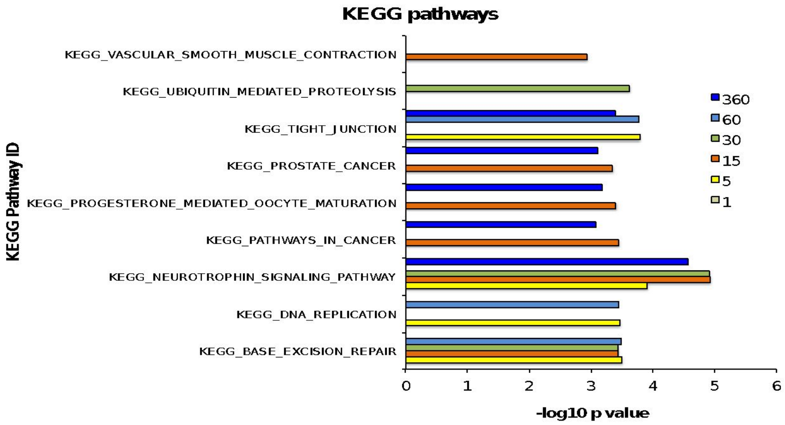
KEGG pathways
KEGG Pathway ID
KEGG_VASCULAR_SMOOTH_MUSCLE_CONTRACTION
KEGG_UBIQUITIN_MEDIATED_PROTEOLYSIS
KEGG_TIGHT_JUNCTION
KEGG_PROSTATE_CANCER
KEGG_PROGESTERONE_MEDIATED_OOCYTE_MATURATION
KEGG_PATHWAYS_IN_CANCER
KEGG_NEUROTROPHIN_SIGNALING_PATHWAY
KEGG_DNA_REPLICATION
KEGG_BASE_EXCISION_REPAIR
360
60
30
15
5
1
-log10 p value

D

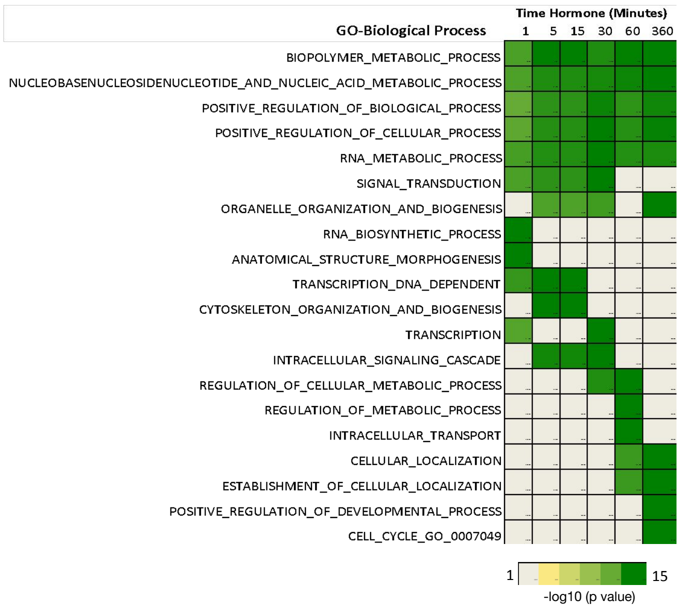
GO-Biological Process
Time Hormone (Minutes)
1 5 15 30 60 360
BIOPOLYMER_METABOLIC_PROCESS
NUCLEOBASENUCLEOSIDENUCLEOTIDE_AND_NUCLEIC_ACID_METABOLIC_PROCESS
POSITIVE_REGULATION_OF_BIOLOGICAL_PROCESS
POSITIVE_REGULATION_OF_CELLULAR_PROCESS
RNA_METABOLIC_PROCESS
SIGNAL_TRANSDUCTION
ORGANELLE_ORGANIZATION_AND_BIOGENESIS
RNA_BIOSYNTHETIC_PROCESS
ANATOMICAL_STRUCTURE_MORPHOGENESIS
TRANSCRIPTION_DNA_DEPENDENT
CYTOSKELETON_ORGANIZATION_AND_BIOGENESIS
TRANSCRIPTION
INTRACELLULAR_SIGNALING_CASCADE
REGULATION_OF_CELLULAR_METABOLIC_PROCESS
REGULATION_OF_METABOLIC_PROCESS
INTRACELLULAR_TRANSPORT
CELLULAR_LOCALIZATION
ESTABLISHMENT_OF_CELLULAR_LOCALIZATION
POSITIVE_REGULATION_OF_DEVELOPMENTAL_PROCESS
CELL_CYCLE_GO_0007049
1
15
-log10 (p value)

E

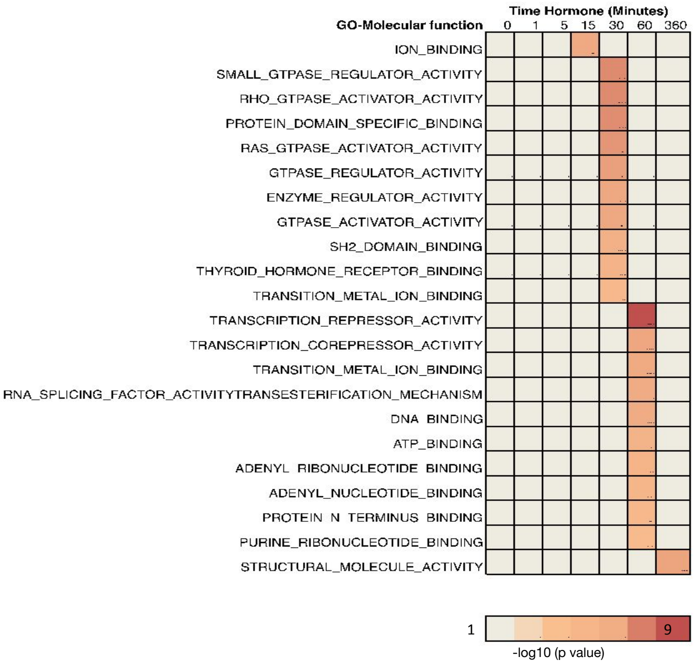
Time Hormone (Minutes)
GO-Molecular function
0 1 5 15 30 60 360
ION_BINDING
SMALL_GTPASE_REGULATOR_ACTIVITY
RHO_GTPASE_ACTIVATOR_ACTIVITY
PROTEIN_DOMAIN_SPECIFIC_BINDING
RAS_GTPASE_ACTIVATOR_ACTIVITY
GTPASE_REGULATOR_ACTIVITY
ENZYME_REGULATOR_ACTIVITY
GTPASE_ACTIVATOR_ACTIVITY
SH2_DOMAIN_BINDING
THYROID_HORMONE_RECEPTOR_BINDING
TRANSITION_METAL_ION_BINDING
TRANSCRIPTION_REPRESSOR_ACTIVITY
TRANSCRIPTION_COREPRESSOR_ACTIVITY
TRANSITION_METAL_ION_BINDING
RNA_SPLICING_FACTOR_ACTIVITYTRANSESTERIFICATION_MECHANISM
DNA_BINDING
ATP_BINDING
ADENYL_RIBONUCLEOTIDE_BINDING
ADENYL_NUCLEOTIDE_BINDING
PROTEIN_N_TERMINUS_BINDING
PURINE_RIBONUCLEOTIDE_BINDING
STRUCTURAL_MOLECULE_ACTIVITY
1
9
-log10 (p value)
