# Supplementary material for: Global signalling network analysis of luminal T47D breast cancer cells in response to progesterone
Source: Front Endocrinol (Lausanne). 2022 Aug 11;13:888802. doi: 10.3389/fendo.2022.888802 (PMC9403329; doi:10.3389/fendo.2022.888802)
Supplement: Supplementary Figure 4 — Combining Antibody Array and Phosphoproteomic LC-MS-MS datasets. (A) Schematic representation showing the methodology and overlap combining antibody array and LC-MS-MS datasets. (B) PCA analysis of phosphorylation datasets. (C) Number of phosphosites identified per protein, the names of proteins showing multiple sites per protein are highlighted. (D) Venn diagram showing the overlap of phosphosites per time point. (E) Up and down regulated phosphorylation sites identified per time point. (F) Phosphorylation levels of the proteins identified as significantly regulated after hormone located within the mitochondria. (G) Analysis of the number of functions to which each unique protein was assigned (H) Venn diagram showing the overlap of protein functional class; Enzymes, Structural protein, Membrane-cell-cell contact, protein modulators and proteins with nucleic acid binding capacities. [file DataSheet_2.pdf]

Fig. S4.

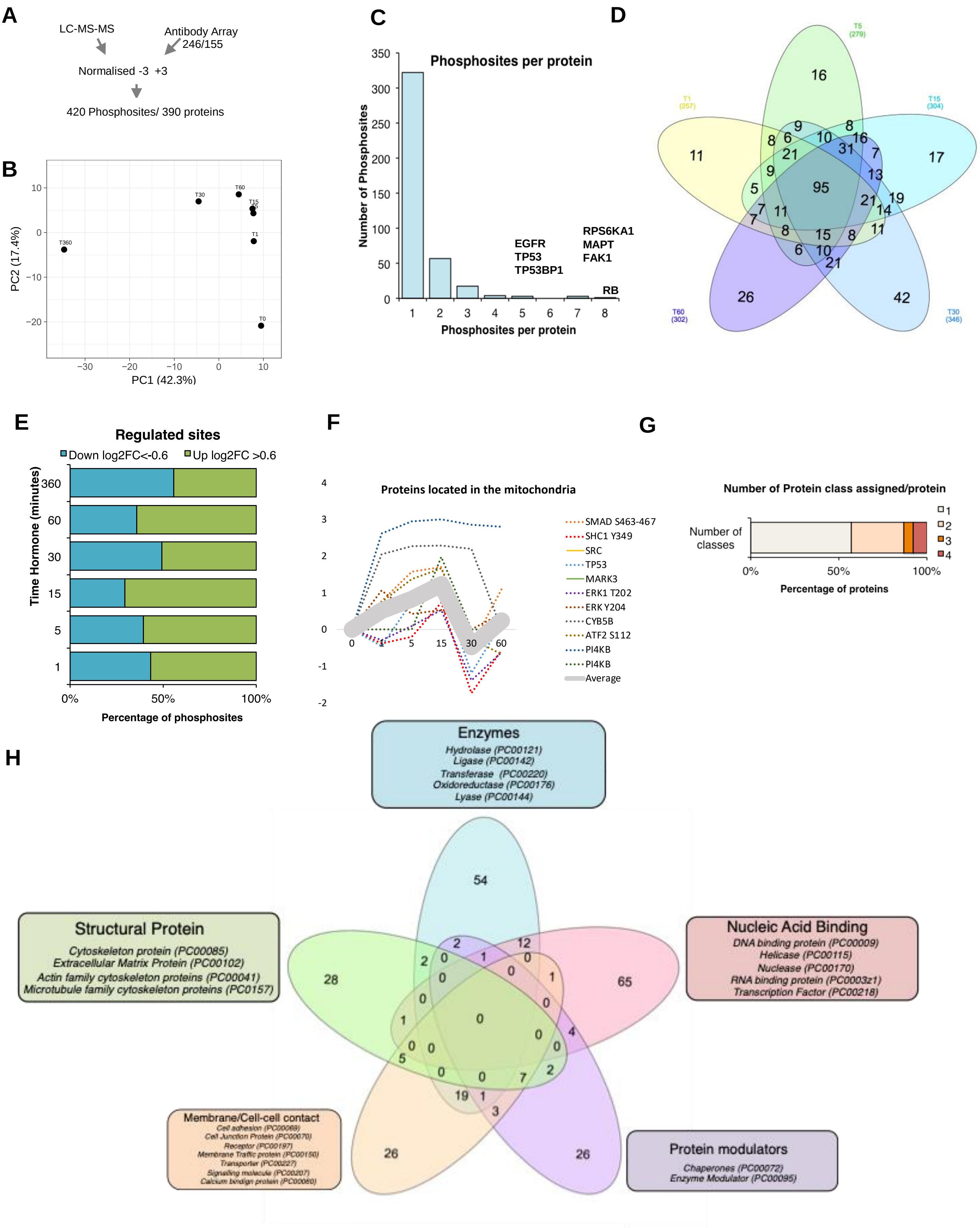
A
LC-MS-MS
Antibody Array
246/155
Normalised -3 +3
420 Phosphosites/ 390 proteins
B
PC2 (17.4%)
PC1 (42.3%)
T0
T1
T15
T30
T60
T360
C
Phosphosites per protein
Number of Phosphosites
EGFR
TP53
TP53BP1
RPS6KA1
MAPT
FAK1
RB
D
T1 (257)
T5 (279)
T15 (304)
T30 (346)
T60 (302)
E
Regulated sites
Down log2FC<-0.6
Up log2FC >0.6
Time Hormone (minutes)
Percentage of phosphosites
F
Proteins located in the mitochondria
SMAD S463-467
SHC1 Y349
SRC
TP53
MARK3
ERK1 T202
ERK Y204
CYB5B
ATF2 S112
PI4KB
PI4KB
Average
G
Number of Protein class assigned/protein
Number of classes
Percentage of proteins
H
Enzymes
Hydrolase (PC00121)
Ligase (PC00142)
Transferase (PC00220)
Oxidoreductase (PC00176)
Lyase (PC00144)
Structural Protein
Cytoskeleton protein (PC00085)
Extracellular Matrix Protein (PC00102)
Actin family cytoskeleton proteins (PC00041)
Microtubule family cytoskeleton proteins (PC0157)
Nucleic Acid Binding
DNA binding protein (PC00009)
Helicase (PC00115)
Nuclease (PC00170)
RNA binding protein (PC0003z1)
Transcription Factor (PC00218)
Membrane/Cell-cell contact
Protein modulators
Chaperones (PC00072)
Enzyme Modulator (PC00095)

Fig. S5

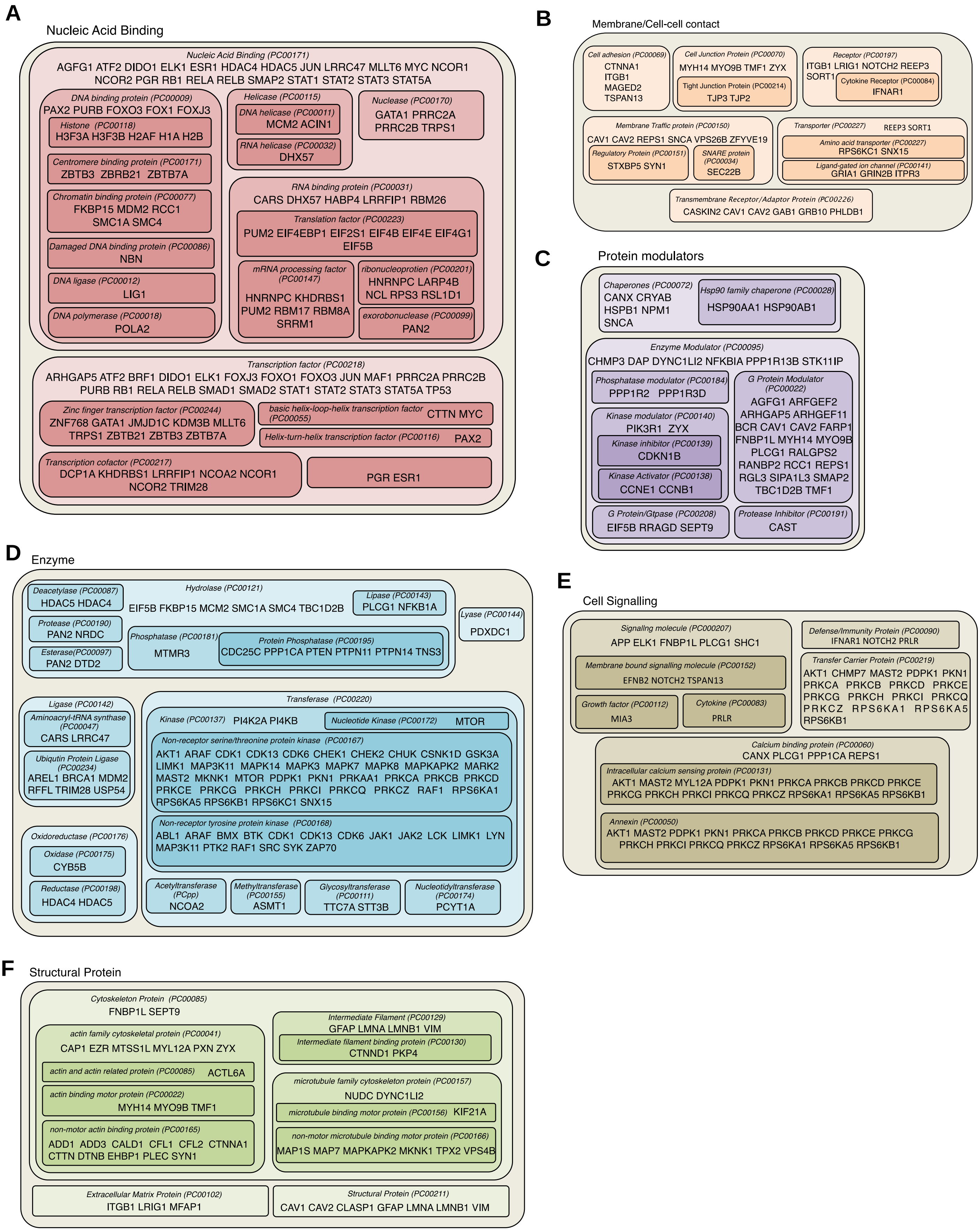
A
Nucleic Acid Binding
Nucleic Acid Binding (PC00171)
AGFG1 ATF2 DIDO1 ELK1 ESR1 HDAC4 HDAC5 JUN LRRC47 MLLT6 MYC NCOR1 NCOR2 PGR RB1 RELA RELB SMAP2 STAT1 STAT2 STAT3 STAT5A
DNA binding protein (PC00009)
PAX2 PURB FOXO3 FOX1 FOXJ3
Histone (PC00118)
H3F3A H3F3B H2AF H1A H2B
Centromere binding protein (PC00171)
ZBTB3 ZBRB21 ZBTB7A
Chromatin binding protein (PC00077)
FKBP15 MDM2 RCC1 SMC1A SMC4
Damaged DNA binding protein (PC00086)
NBN
DNA ligase (PC00012)
LIG1
DNA polymerase (PC00018)
POLA2
Helicase (PC00115)
DNA helicase (PC00011)
MCM2 ACIN1
RNA helicase (PC00032)
DHX57
Nuclease (PC00170)
GATA1 PRRC2A PRRC2B TRPS1
RNA binding protein (PC00031)
CARS DHX57 HABP4 LRRFIP1 RBM26
Translation factor (PC00223)
PUM2 EIF4EBP1 EIF2S1 EIF4B EIF4E EIF4G1 EIF5B
mRNA processing factor (PC00147)
HNRNPC KHDRBS1 PUM2 RBM17 RBM8A SRRM1
ribonucleoprotien (PC00201)
HNRNPC LARP4B NCL RPS3 RSL1D1
exorobonuclease (PC00099)
PAN2
Transcription factor (PC00218)
ARHGAP5 ATF2 BRF1 DIDO1 ELK1 FOXJ3 FOXO1 FOXO3 JUN MAF1 PRRC2A PRRC2B PURB RB1 RELA RELB SMAD1 SMAD2 STAT1 STAT2 STAT3 STAT5A TP53
Zinc finger transcription factor (PC00244)
ZNF768 GATA1 JMJD1C KDM3B MLLT6 TRPS1 ZBTB21 ZBTB3 ZBTB7A
basic helix-loop-helix transcription factor (PC00055)
CTTN MYC
Helix-turn-helix transcription factor (PC00116)
PAX2
Transcription cofactor (PC00217)
DCP1A KHDRBS1 LRRFIP1 NCOA2 NCOR1 NCOR2 TRIM28
PGR ESR1
B
Membrane/Cell-cell contact
Cell adhesion (PC00069)
CTNNA1 ITGB1 MAGED2 TSPAN13
Cell Junction Protein (PC00070)
MYH14 MYO9B TMF1 ZYX
Tight Junction Protein (PC00214)
TJP3 TJP2
Receptor (PC00197)
ITGB1 LRIG1 NOTCH2 REEP3 SORT1
Cytokine Receptor (PC00084)
IFNAR1
Membrane Traffic protein (PC00150)
CAV1 CAV2 REPS1 SNCA VPS26B ZFYVE19
Regulatory Protein (PC00151)
STXBP5 SYN1
SNARE protein (PC00034)
SEC22B
Transporter (PC00227)
REEP3 SORT1
Amino acid transporter (PC00227)
RPS6KC1 SNX15
Ligand-gated ion channel (PC00141)
GRIA1 GRIN2B ITPR3
Transmembrane Receptor/Adaptor Protein (PC00226)
CASKIN2 CAV1 CAV2 GAB1 GRB10 PHLDB1
C
Protein modulators
Chaperones (PC00072)
CANX CRYAB HSPB1 NPM1 SNCA
Hsp90 family chaperone (PC00028)
HSP90AA1 HSP90AB1
Enzyme Modulator (PC00095)
CHMP3 DAP DYNC1LI2 NFKBIA PPP1R13B STK11IP
Phosphatase modulator (PC00184)
PPP1R2 PPP1R3D
G Protein Modulator (PC00022)
AGFG1 ARFGEF2 ARHGAP5 ARHGEF11 BCR CAV1 CAV2 FARP1 FNBP1L MYH14 MYO9B PLCG1 RALGPS2 RANBP2 RCC1 REPS1 RGL3 SIPA1L3 SMAP2 TBC1D2B TMF1
Kinase modulator (PC00140)
PIK3R1 ZYX
Kinase inhibitor (PC00139)
CDKN1B
Kinase Activator (PC00138)
CCNE1 CCNB1
G Protein/Gtpase (PC00208)
EIF5B RRAGD SEPT9
Protease Inhibitor (PC00191)
CAST
D
Enzyme
Deacetylase (PC00087)
HDAC5 HDAC4
Hydrolase (PC00121)
EIF5B FKBP15 MCM2 SMC1A SMC4 TBC1D2B
Lipase (PC00143)
PLCG1 NFKB1A
Protease (PC00190)
PAN2 NRDC
Esterase(PC00097)
PAN2 DTD2
Phosphatase (PC00181)
MTMR3
Protein Phosphatase (PC00195)
CDC25C PPP1CA PTEN PTPN11 PTPN14 TNS3
Lyase (PC00144)
PDXDC1
Ligase (PC00142)
Aminoacryl-tRNA synthase (PC00047)
CARS LRRC47
Ubiqutin Protein Ligase (PC00234)
AREL1 BRCA1 MDM2 RFFL TRIM28 USP54
Oxidoreductase (PC00176)
Oxidase (PC00175)
CYB5B
Reductase (PC00198)
HDAC4 HDAC5
Transferase (PC00220)
Kinase (PC00137) PI4K2A PI4KB
Nucleotide Kinase (PC00172) MTOR
Non-receptor serine/threonine protein kinase (PC00167)
AKT1 ARAF CDK1 CDK13 CDK6 CHEK1 CHEK2 CHUK CSNK1D GSK3A LIMK1 MAP3K11 MAPK14 MAPK3 MAPK7 MAPK8 MAPKAPK2 MARK2 MAST2 MKNK1 MTOR PDPK1 PKN1 PRKAA1 PRKCA PRKCB PRKCD PRKCE PRKCG PRKCH PRKCI PRKCQ PRKCZ RAF1 RPS6KA1 RPS6KA5 RPS6KB1 RPS6KC1 SNX15
Non-receptor tyrosine protein kinase (PC00168)
ABL1 ARAF BMX BTK CDK1 CDK13 CDK6 JAK1 JAK2 LCK LIMK1 LYN MAP3K11 PTK2 RAF1 SRC SYK ZAP70
Acetyltransferase (PCpp)
NCOA2
Methyltransferase (PC00155)
ASMT1
Glycosyltransferase (PC00111)
TTC7A STT3B
Nucleotidyltransferase (PC00174)
PCYT1A
E
Cell Signalling
Signallng molecule (PC000207)
APP ELK1 FNBP1L PLCG1 SHC1
Membrane bound signalling molecule (PC00152)
EFNB2 NOTCH2 TSPAN13
Growth factor (PC00112)
MIA3
Cytokine (PC00083)
PRLR
Defense/Immunity Protein (PC00090)
IFNAR1 NOTCH2 PRLR
Transfer Carrier Protein (PC00219)
AKT1 CHMP7 MAST2 PDPK1 PKN1 PRKCA PRKCB PRKCD PRKCE PRKCG PRKCH PRKCI PRKCQ PRKCZ RPS6KA1 RPS6KA5 RPS6KB1
Calcium binding protein (PC00060)
CANX PLCG1 PPP1CA REPS1
Intracellular calcium sensing protein (PC00131)
AKT1 MAST2 MYL12A PDPK1 PKN1 PRKCA PRKCB PRKCD PRKCE PRKCG PRKCH PRKCI PRKCQ PRKCZ RPS6KA1 RPS6KA5 RPS6KB1
Annexin (PC00050)
AKT1 MAST2 PDPK1 PKN1 PRKCA PRKCB PRKCD PRKCE PRKCG PRKCH PRKCI PRKCQ PRKCZ RPS6KA1 RPS6KA5 RPS6KB1
F
Structural Protein
Cytoskeleton Protein (PC00085)
FNBP1L SEPT9
actin family cytoskeletal protein (PC00041)
CAP1 EZR MTSS1L MYL12A PXN ZYX
actin and actin related protein (PC00085) ACTL6A
actin binding motor protein (PC00022)
MYH14 MYO9B TMF1
non-motor actin binding protein (PC00165)
ADD1 ADD3 CALD1 CFL1 CFL2 CTNNA1 CTTN DTNB EHBP1 PLEC SYN1
Intermediate Filament (PC00129)
GFAP LMNA LMNB1 VIM
Intermediate filament binding protein (PC00130)
CTNND1 PKP4
microtubule family cytoskeleton protein (PC00157)
NUDC DYNC1LI2
microtubule binding motor protein (PC00156) KIF21A
non-motor microtubule binding motor protein (PC00166)
MAP1S MAP7 MAPKAPK2 MKNK1 TPX2 VPS4B
Extracellular Matrix Protein (PC00102)
ITGB1 LRIG1 MFAP1
Structural Protein (PC00211)
CAV1 CAV2 CLASP1 GFAP LMNA LMNB1 VIM

Fig. S6

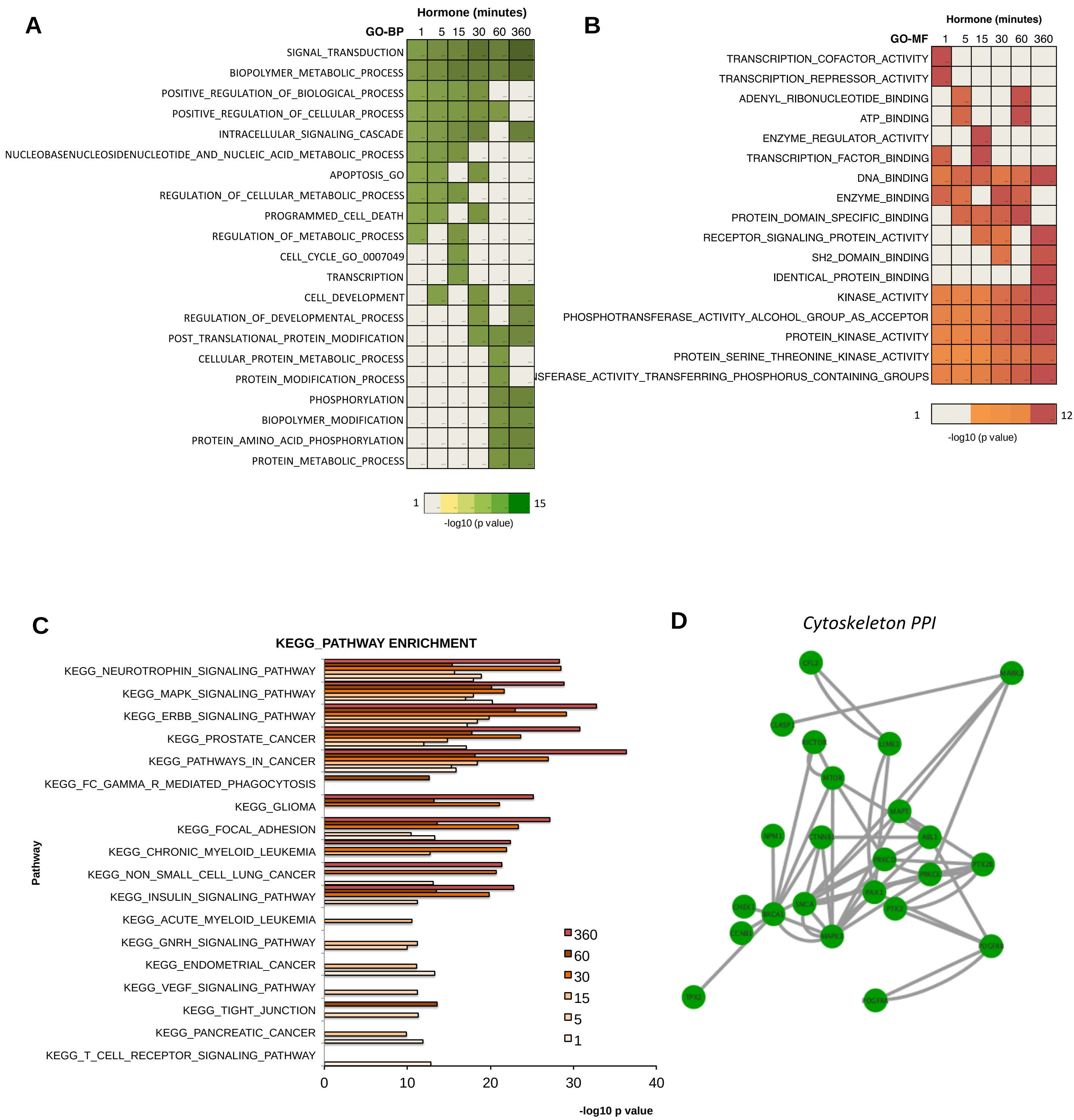
A
Hormone (minutes)
GO-BP
1 5 15 30 60 360
SIGNAL_TRANSDUCTION
BIOPOLYMER_METABOLIC_PROCESS
POSITIVE_REGULATION_OF_BIOLOGICAL_PROCESS
POSITIVE_REGULATION_OF_CELLULAR_PROCESS
INTRACELLULAR_SIGNALING_CASCADE
NUCLEOBASENUCLEOSIDENUCLEOTIDE_AND_NUCLEIC_ACID_METABOLIC_PROCESS
APOPTOSIS_GO
REGULATION_OF_CELLULAR_METABOLIC_PROCESS
PROGRAMMED_CELL_DEATH
REGULATION_OF_METABOLIC_PROCESS
CELL_CYCLE_GO_0007049
TRANSCRIPTION
CELL_DEVELOPMENT
REGULATION_OF_DEVELOPMENTAL_PROCESS
POST_TRANSLATIONAL_PROTEIN_MODIFICATION
CELLULAR_PROTEIN_METABOLIC_PROCESS
PROTEIN_MODIFICATION_PROCESS
PHOSPHORYLATION
BIOPOLYMER_MODIFICATION
PROTEIN_AMINO_ACID_PHOSPHORYLATION
PROTEIN_METABOLIC_PROCESS
1
15
-log10 (p value)
B
Hormone (minutes)
GO-MF
1 5 15 30 60 360
TRANSCRIPTION_COFACTOR_ACTIVITY
TRANSCRIPTION_REPRESSOR_ACTIVITY
ADENYL_RIBONUCLEOTIDE_BINDING
ATP_BINDING
ENZYME_REGULATOR_ACTIVITY
TRANSCRIPTION_FACTOR_BINDING
DNA_BINDING
ENZYME_BINDING
PROTEIN_DOMAIN_SPECIFIC_BINDING
RECEPTOR_SIGNALING_PROTEIN_ACTIVITY
SH2_DOMAIN_BINDING
IDENTICAL_PROTEIN_BINDING
KINASE_ACTIVITY
PHOSPHOTRANSFERASE_ACTIVITY_ALCOHOL_GROUP_AS_ACCEPTOR
PROTEIN_KINASE_ACTIVITY
PROTEIN_SERINE_THREONINE_KINASE_ACTIVITY
ISFERASE_ACTIVITY_TRANSFERRING_PHOSPHORUS_CONTAINING_GROUPS
1
12
-log10 (p value)
C
KEGG_PATHWAY ENRICHMENT
KEGG_NEUROTROPHIN_SIGNALING_PATHWAY
KEGG_MAPK_SIGNALING_PATHWAY
KEGG_ERBB_SIGNALING_PATHWAY
KEGG_PROSTATE_CANCER
KEGG_PATHWAYS_IN_CANCER
KEGG_FC_GAMMA_R_MEDIATED_PHAGOCYTOSIS
KEGG_GLIOMA
KEGG_FOCAL_ADHESION
KEGG_CHRONIC_MYELOID_LEUKEMIA
KEGG_NON_SMALL_CELL_LUNG_CANCER
KEGG_INSULIN_SIGNALING_PATHWAY
KEGG_ACUTE_MYELOID_LEUKEMIA
KEGG_GNRH_SIGNALING_PATHWAY
KEGG_ENDOMETRIAL_CANCER
KEGG_VEGF_SIGNALING_PATHWAY
KEGG_TIGHT_JUNCTION
KEGG_PANCREATIC_CANCER
KEGG_T_CELL_RECEPTOR_SIGNALING_PATHWAY
Pathway
360
60
30
15
5
1
0
10
20
30
40
-log10 p value
D
Cytoskeleton PPI

Fig. S7

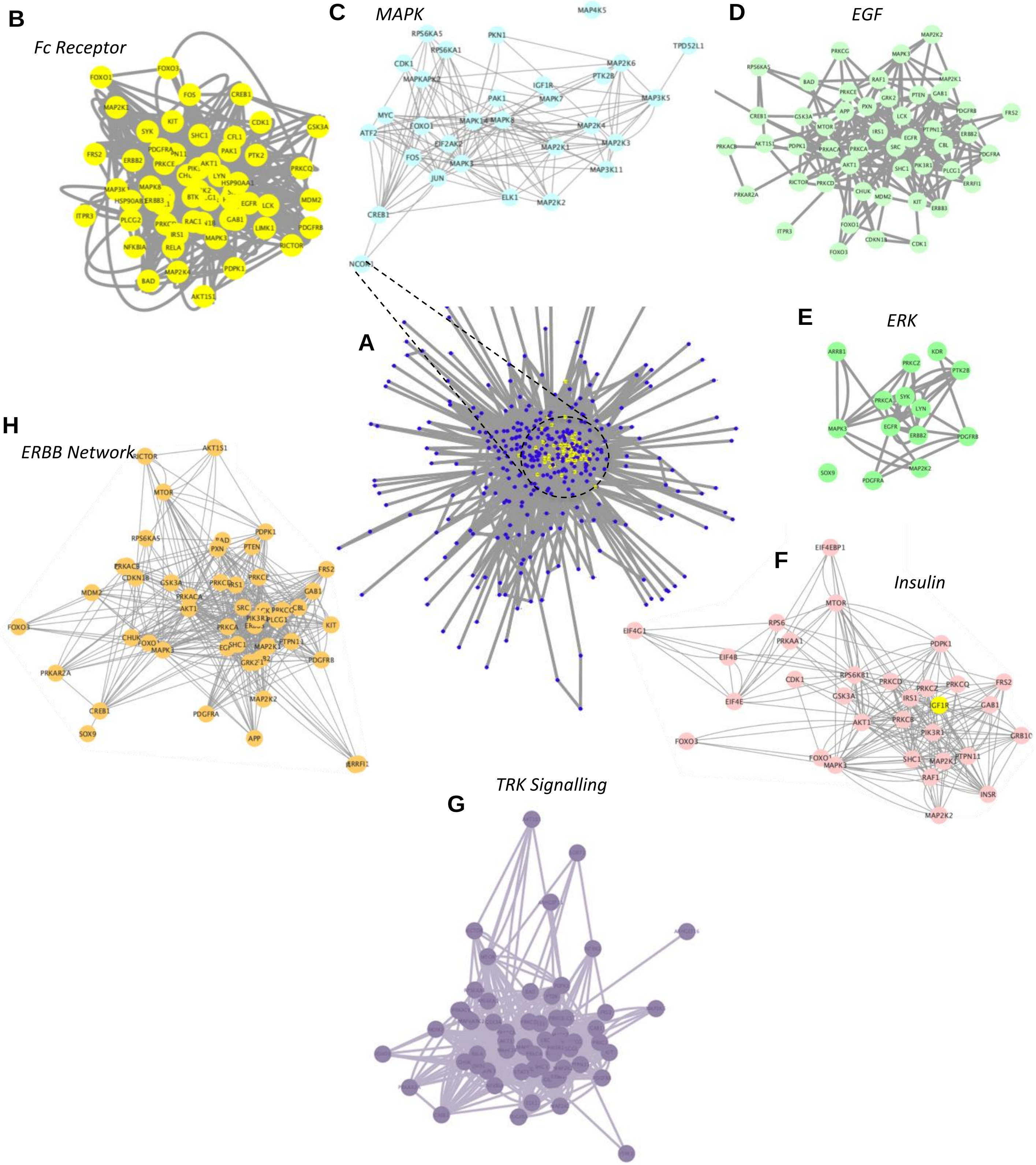
B
Fc Receptor
C
MAPK
D
EGF
A
E
ERK
H
ERBB Network
F
Insulin
TRK Signalling
G
