# Supplementary material for: Global signalling network analysis of luminal T47D breast cancer cells in response to progesterone
Source: Front Endocrinol (Lausanne). 2022 Aug 11;13:888802. doi: 10.3389/fendo.2022.888802 (PMC9403329; doi:10.3389/fendo.2022.888802)
Supplement: Network Session 1 — Edge directed PKN network was manually curated from the literature. Annotated phosphorylation events, interactions, dissociations and cellular compartment are indicated. [file DataSheet_4.docx]

**Supplementary Figure Legends**

**Fig. S1. Prior Knowledge Network (PKN) Progesterone Signalling.**

**A)** Edge directed PKN network was manually curated from the literature. Annotated phosphorylation events, interactions, dissociations and cellular compartment are indicated. Network is available as a cytoscape network session (cys) or interaction (sys) file containing references for all edges present as shown in B) (See Supplementary File Network 1).

**Fig. S2. Antibody Array controls and data analysis.**

**A)** Schematic indicating the experimental procedure, quality control checks, and filtering applied to the antibody array experiments. **B)** Number of phosphosites identified per protein. Tau, PTK2, RPS6KA1 and RB1 are highlighted as they have multiple sites identified**. C)** Functional classification of the proteins identified as significantly phosphorylated at each time point **D)** KEGG pathway analysis, showing significant pathways (-log_10_ p-value) at each time point. **E)** Heatmap representation of GO biological process data, showing significant (-log_10_ p-value) processes at each time point. **F)** Heatmap representation of GO molecular function data, showing significant (-log_10_ p-value) functions at each time point.

**Fig. S3. Phosphoproteomic data acquisition and controls.**

**A)** Correlation of triplicate samples from each of the time points. **B)** Number of phosphosites identified per protein, the proteins showing multiple sites per protein are highlighted. **C)**  KEGG pathway analysis, showing significant pathways (-log_10_ p-value) at each time point. **D)** Heatmap representation of GO-biological process data, showing significant (-log_10_ p-value) processes at each time point. **E)** Heatmap representation of GO-molecular function data, showing significant (-log_10_ p-value) functions per time point.

**Fig S4. Combining Antibody Array and Phosphoproteomic LC-MS-MS datasets.**

**A)** Schematic representation showing the methodology and overlap combining antibody array and LC-MS-MS datasets. **B)** PCA analysis of phosphorylation datasets. **C)** Number of phosphosites identified per protein, the names of proteins showing multiple sites per protein are highlighted. **D)** Venn diagram showing the overlap of phosphosites per time point. **E)** Up and down regulated phosphorylation sites identified per time point. **F)** Phosphorylation levels of the proteins identified as significantly regulated after hormone located within the mitochondria. **G)** Analysis of the number of functions to which each unique protein was assigned **H)** Venn diagram showing the overlap of protein functional class; Enzymes, Structural protein, Membrane-cell-cell contact, protein modulators and proteins with nucleic acid binding capacities.

**Fig S5. Functional Analysis of Proteins Identified.**

All proteins were assigned one or more function based on the GSEA database. Both Parent (outside/title), and children (within) are shown for each class and the proteins identified within that sub-group are shown. Nucleic acid binding **(A),** Membrane/Cell-cell contact **(B),** Protein Modulators **(C),** Enzymes **(D),** Cell signalling **(E),** and Structural proteins **(F).**

**Fig S6. Gene Ontology and Pathway analysis of combined dataset.**

**A)** Heatmap representation of GO biological process data, showing significant (-log_10_ p-value) biological processes enrichment based on the protein phosphorylation at each time point. **B)** Heatmap representation of GO molecular function enrichment, showing significant (-log_10_ p-value) functions at each time point following hormone. **C)** KEGG pathway analysis, showing significant pathways (-log_10_ p-value) enriched at each time point following hormone exposure. **D)** Protein protein interaction (PPI) network generated using proteins identified as phosphorylated following hormone and were assigned as cytoskeleton located.

**Fig S7. Pathway Network Generation in Breast Cancer cells in response to Hormone.**

**A)** Protein protein interaction (PPI) network was generated using a full phosphorylation dataset encompassing 321 proteins (Supplementary Material Network session 2) in Cytoscape using Genemania^TM^ only considering protein-protein interactions with experimental evidence (Supp. Materials and methods), each node represents and individual protein and interactions are represented by edges. Functional analysis was carried out to identify key pathways enriched within the full network (Full list Supplementary Table 16). Individual networks were generated from each function individually and are available within additional Network session 2. Graphs of several pathways determined to be enriched within the dataset are shown **B)** Fc receptor, **C)** MAPK, **D)** EGF **E)** ERK **F)** Insulin, **G)** TRK signalling, **H)** ERBB.

**Supplementary Table Legends**

**Supplementary Table S1**

Uniprot IDs of phosphorylated proteins identified in response to hormone. Time after hormone (minutes), data is normalised 0-1 row maximum and minimum.

**Supplementary Table S2**

KEGG pathway enrichment; the pathway term, p value and the proteins associated with the pathway are shown.

**Supplementary Table S3**

Cellular component enrichment analysis of phosphorylated proteins. The time after hormone in which they peak, the adjusted p value, and proteins associated with each specific cellular component are given.

**Supplementary Table S4**

Gene Ontology Biological Process enrichment analysis of phosphorylated proteins. The cluster in which the term is enriched, the adjusted p value, and proteins associated with each specific biological process are given.

**Supplementary Table S5**

Gene Ontology Molecular Function enrichment analysis of phosphorylated proteins. The cluster in which the term is enriched, the adjusted p value, and proteins associated with each specific molecular function are given.

**Supplementary Table S6**

Corum enrichment analysis of phosphorylated proteins. The p-value, and proteins associated with each complex are given.

**Supplementary Table S7**

Cellular component enrichment analysis of phosphorylated and PARylated proteins. The adjusted p value, and proteins associated with each specific cellular component are given.

**Supplementary Table S8**

Corum enrichment analysis of phosphorylated and PARylated proteins. The p-value, and proteins associated with each complex are given, phosphorylated proteins are highlighted in yellow.

**Supplementary Table S9**

Genemania analysis of phosphorylated proteins, all protein IDs are listed along with the GO: IDs for which they are associated.

**Supplementary Table S10**

Genemania analysis of phosphorylated proteins, the pathways enriched in Network 2 are shown. The q value and the number of occurrences in the network versus the occurrences in the Network are shown.

**Additional Files**

**Network Session 1**

Edge directed PKN network was manually curated from the literature. Annotated phosphorylation events, interactions, dissociations and cellular compartment are indicated.

**Network Session 2**

Protein protein interaction (PPI) network was generated using a full phosphorylation dataset encompassing 321 proteins in Cytoscape using Genemania^TM^ only considering protein-protein interactions with experimental evidence each node represents and individual protein and interactions are represented by edges. Functional analysis was carried out to identify key pathways enriched within the full network. Individual networks were generated from each function individually and are available as unique networks within the Network session.
